# Eosinophilic inflammation in hereditary angioedema: a single-center real-world retrospective chart review study

**DOI:** 10.3389/fimmu.2026.1754405

**Published:** 2026-02-17

**Authors:** Katharina Boch, Ralf J. Ludwig, Dagmar von Bubnoff, Andreas Recke

**Affiliations:** 1 Department of Dermatology, University Hospital, LMU Munich, Munich, Germany; 2 Department of Dermatology, University of Lübeck, Lübeck, Germany; 3 Lübeck Institute of Experimental Dermatology, University of Lübeck, Lübeck, Germany

**Keywords:** biomarker, eosinophilic cationic protein, eosinophilic inflammation, hereditary angioedema, subclinical inflammation

## Abstract

**Background:**

Hereditary angioedema (HAE) is a rare genetic disorder characterized by recurrent, unpredictable swelling attacks primarily driven by bradykinin-mediated vascular permeability. However, additional inflammatory mechanisms may contribute to disease heterogeneity. During routine diagnostics, we observed elevated serum eosinophil cationic protein (ECP) levels in HAE patients, suggesting increased eosinophil activation. To date, eosinophil involvement in HAE has not been systematically investigated, this study aimed to validate clinical observations and explore a potential link between bradykinin signaling and eosinophilic inflammation.

**Methods:**

We retrospectively analyzed data from 48 patients with confirmed HAE (32 HAE type I/II, 16 HAE with normal C1-INH) and 1,880 control patients treated at a tertiary university allergy and angioedema referral center. Using causal-inference Bayesian multilevel regression with bias-breaking post-stratification and propensity-score inverse probability weighting, we estimated the effect of HAE on serum ECP levels and absolute eosinophil counts while adjusting for age, sex, season, and allergic comorbidities.

**Results:**

HAE was associated with a 1.52-fold average increase in serum ECP levels (most conservative 95% credibility interval: 1.24–1.90; Bayesian p = 0.00088), consistent across all modeling specifications. Absolute eosinophil counts were not elevated, indicating enhanced eosinophil activation independent of cell number.

**Conclusions:**

Patients with HAE show biochemical evidence of increased eosinophil activation, suggesting a previously unrecognized inflammatory component beyond bradykinin-driven edema formation. Further studies should clarify clinical implications and the potential contribution to comorbidities and phenotypic variability.

## Introduction

Hereditary angioedema (HAE) is a rare inherited disorder characterized by recurrent and unpredictable swelling attacks (1, 2). Attacks affecting the tongue or larynx may cause life-threatening upper-airway obstruction, whereas those involving the intestinal tract are typically accompanied by severe abdominal pain and distension (1, 2). In about 80% of cases, HAE results from a heterozygous autosomal mutation in *SERPING1*, leading to quantitative or functional deficiency of the C1 esterase inhibitor (C1-INH; HAE-C1INH) (1, 3). The lack of functional C1-INH causes excessive generation of bradykinin, which increases vascular permeability through activation of the bradykinin B2 receptor, ultimately resulting in the clinical manifestation of angioedema (1).

Besides *SERPING1* mutations, other genetic variants have been identified in patients with normal C1-INH concentrations (HAE-nC1INH). In the U.S., the prevalence of this HAE type is estimated at approximately 3.7 per million, whereas HAE-C1INH has an estimated prevalence of 10–15 per million, depending on the population studied (3, 4). These include pathogenic variants in *F12*, *ANGPT1*, *PLG*, *KNG1*, *MYOF*, and *HS3ST6* (5–10). More recently, *CPN1* and *DAB2IP* have also been implicated (11, 12). Nevertheless, the causative defect remains unknown in a substantial fraction of cases classified as HAE-nC1INH (2), with reported proportions strongly depending on case definitions and genetic testing strategies. In cohorts applying a broad phenotypic definition of angioedema without wheals, up to 87.9% of patients lacked an identifiable causative gene variant (13). Although the precise mechanisms of HAE-nC1INH are not fully understood, bradykinin appears to play a central pathogenic role in most variants, except those associated with *ANGPT1* and *MYOF* (10, 14). This is supported by findings from Bova et al. showing impaired regulation of the contact system in HAE-nC1INH, leading to increased susceptibility to uncontrolled bradykinin generation (15).

Despite these advances in understanding the genetic and molecular basis of HAE, translating pathophysiological insights into clinically useful biomarkers has remained challenging. Current diagnostic guidelines provide valuable clinical criteria, but genetic testing remains the only definitive tool to confirm HAE. Novel biomarkers could help to fill this diagnostic gap – by distinguishing HAE from more common forms of angioedema, enabling monitoring of disease activity, and improving our understanding of the underlying disease mechanisms (1, 16, 17).

While most proposed biomarkers focus on components of the contact and complement systems (16), routine laboratory testing may also reveal inflammatory signals beyond these established pathways. Through routine clinical observation, we noted consistently elevated levels of eosinophil cationic protein (ECP) in patients with HAE, irrespective of whether the condition was associated with C1-INH deficiency or normal C1-INH levels. As serum ECP measurement is routinely used as a marker of eosinophilic inflammation, this unexpected finding prompted us to systematically investigate ECP levels in HAE by analyzing routinely collected laboratory data from patients treated at our institution.

Eosinophilic cationic protein (ECP), also known as ribonuclease 3 (*RNASE3*), possesses antimicrobial and anthelmintic properties and is predominantly expressed and released by eosinophil granulocytes (18, 19). Measurement of serum ECP has proven useful for monitoring type 2 inflammatory diseases such as atopic dermatitis, bronchial asthma, and chronic rhinosinusitis with nasal polyps (20–22).

To date, eosinophil activation has not been systematically investigated in hereditary angioedema, although inflammatory processes beyond the contact system have been increasingly discussed (17, 23). Notably, episodic angioedema can also occur in hypereosinophilic syndromes, commonly known as Gleich’s syndrome. This disorder is characterized by markedly elevated eosinophil counts and extensive eosinophilic tissue infiltration (24–26). Although Gleich’s syndrome is pathogenetically distinct from HAE, it illustrates that angioedema as a clinical phenotype can be associated with pronounced eosinophil activation.

An in-depth literature search revealed only a few reports mentioning eosinophils in the context of HAE (23, 27, 28), yet none providing direct quantitative or mechanistic analyses of eosinophil activation. Consequently, detailed insights into the potential interplay between eosinophils and HAE pathophysiology remain lacking. Nevertheless, emerging evidence from hypothesis-free approaches suggests that inflammatory pathways beyond the classical contact system may indeed be involved in HAE. In line with this, recent multi-omics analyses have identified increased galectin-3 levels in samples from patients with HAE-nC1INH, a lectin known to play a critical role in eosinophil recruitment and allergic airway inflammation (28). Notably, elevated galectin-3 concentrations were also observed in a subset of HAE-C1INH (types I and II) samples in that study. In addition, recent work by Gil-Serrano et al. demonstrated that several systemic inflammatory biomarkers rise during HAE attacks, indicating that inflammatory pathways may be more involved in HAE pathophysiology than previously assumed (17). Interestingly, bradykinin can induce eosinophil migration and elastase release via the bradykinin B1 and B2 receptors (23), suggesting a potential, yet unexplored, mechanistic link between bradykinin signaling and eosinophil activation in HAE.

Clinical attention in hereditary angioedema has traditionally focused on acute swelling attacks. However, such an attack-centered perspective may overlook disease-related processes that persist outside clinically overt episodes. Evidence for ongoing subclinical activity is provided by persistent complement consumption, exemplified by chronically reduced C4 levels even during symptom-free intervals (29), indicating that HAE is characterized by continuous underlying disease activity (15, 23).

In this context, the identification of increased eosinophil activation in HAE patients suggests that inflammatory processes involving immune cell activation and endothelial dysfunction may accompany bradykinin-mediated vascular dysfunction beyond acute edema episodes (23, 27, 28, 30). Appreciating such subclinical disease activity is relevant not only for understanding clinical heterogeneity and eosinophil-associated comorbidities, but also for contextualizing current therapeutic strategies. As highly effective and low-threshold oral on-demand therapies such as sebetralstat and deucrictibant become available and may be sufficient for a subset of HAE patients with lower attack frequencies (31–33), a more comprehensive understanding of disease biology may be essential to inform discussions about the potential role and limitations of purely attack-focused treatment approaches versus long-term prophylaxis.

Against this background, we aimed to determine whether eosinophil activation, reflected by serum ECP levels, represents a previously unrecognized inflammatory component of hereditary angioedema. In this single-center retrospective chart review, we analyzed real-world data on ECP levels in patients with hereditary angioedema (HAE), using other allergic conditions – such as acquired angioedema, rhinitis, sinusitis, atopic dermatitis, urticaria, and allergic asthma – as clinical comparators and adjustment variables. To mitigate selection bias inherent to retrospective designs, we applied causal inference techniques, including post-stratification based on a bias-breaking variable, to obtain more robust estimates of the association between HAE and ECP levels as well as eosinophil counts. On average, ECP levels were 1.52 times higher in HAE patients than in patients with similar clinical profiles. Although ECP is a biomarker with limited individual diagnostic utility due to its susceptibility to pre-analytical variation (34), these findings indicate that HAE is linked with enhanced eosinophil activation, which may contribute to an increased prevalence of eosinophil-associated comorbidities such as asthma or food allergy.

## Methods

### Ethics statement

This study retrospectively collected data from patients and was conducted in accordance with the ethical standards of the institutional review board (vote #21-323) and with the 1964 Helsinki Declaration and its later amendments. Only data from patients who provided general consent for retrospective research were included in the study.

### Generative AI statement

Generative AI tools (ChatGPT (OpenAI), SciSpace, and DeepL) were used to support R coding, data analysis design, literature searches, language translation, and native-language refinement. In addition, large language models were employed for a preliminary internal review of the manuscript to identify inconsistencies, improve clarity, and ensure terminological accuracy. All AI-assisted outputs were critically reviewed and verified by the authors to ensure accuracy, methodological rigor, and adherence to ethical standards.

### Patient cohorts

This study included patients with allergy-related diseases who underwent serum eosinophilic cationic protein (ECP) testing at the University of Lübeck’s allergy outpatient clinic between January 2014 and November 2025. For each ECP test, data were extracted on patient age, sex, test date, eosinophil granulocyte counts (if available), and ICD-10 diagnosis codes from the same quarter.

The primary diagnostic codes of interest were D84.1 (hereditary angioedema, HAE) and T78.3 (angioedema), alongside adjustment variables including J30 (rhinitis), J32 and J33 (sinusitis and nasal polyposis), L20 (atopic dermatitis), L50 (urticaria), D47 (including mastocytosis), and J45 (allergic asthma).

Initial documentation occasionally listed D84.1 as a tentative diagnosis. Therefore, all patients with recorded D84.1 were systematically reviewed. If HAE could not be confirmed based on predefined criteria, the diagnosis was marked as “not present.”

Diagnosis of HAE was based on clinical practice guideline recommendations (1) and internationally consented criteria as described by Buttgereit et al. and Zuraw et al. (35, 36).

For HAE-C1INH (types 1/2), confirmation required repeated laboratory testing demonstrating reduced concentration or function of C1 inhibitor (C1INH). Acquired C1-INH deficiency was excluded based on normal C1q levels and absence of autoimmune/lymphoproliferative disease; hereditary disease was supported by childhood onset, family history and/or pathogenic SERPING1 variant.

HAE (all types) was considered present if the following conditions were fulfilled:

- Recurrent swelling attacks involving the face, extremities, upper airway, genitals, and/or abdominal tract.
- Lack of response to antihistamines, glucocorticoids, and omalizumab, with possible positive response to icatibant.
- Absence of angioedema-inducing medications (ACE inhibitors, NSAIDs, DPP-4 inhibitors, hormonal triggers).
- Positive family history, childhood onset, documented pathogenic variants in established HAE genes (*SERPING1*, *F12*, *PLG*, *ANGPT1*, *KNG1*, *MYOF*, *HS3ST6*, *CPN1*, *DAB2IP*), or clinical improvement under kallikrein or factor XII inhibitors.
- Additional supporting factors included: absence of urticaria, presence of prodromal signs, and response to modern prophylactic therapies (lanadelumab, berotralstat).

Prophylactic treatments were not systematically documented across all records; however, a substantial proportion of ECP measurements in the HAE cohort were obtained during ongoing long-term prophylaxis with berotralstat or lanadelumab as part of routine follow-up care. In contrast, most control patients were sampled at first presentation and were typically not receiving long-term anti-allergic medication at the time of blood collection. Systemic corticosteroids were rarely used in either cohort and were generally reserved for emergency situations; given the elective outpatient setting, relevant corticosteroid exposure at the time of sampling was uncommon. The ECP dataset contained no classical missing data: all extracted ECP measurements had complete accompanying information on age, sex, and test date. Eosinophil counts were simply not ordered at some visits and were therefore absent by design rather than missing. Accordingly, we analyzed (i) the full ECP cohort and (ii) a filtered eosinophil sub-cohort comprising only entries with available eosinophil counts. No imputation was required.

### Sample collection and processing

Blood samples were obtained as part of routine outpatient care during regular clinic hours. For serum preparation, blood was collected using serum tubes with clot activator beads (S-Monovette^®^, 7.5 mL; Sarstedt AG & Co. KG, Nümbrecht, Germany; article no. 01.1601). For complete blood counts including absolute eosinophil numbers, blood was collected using EDTA tubes (S-Monovette^®^ EDTA K3E, 2.7 mL; Sarstedt AG & Co. KG, Nümbrecht, Germany; order no. 04.1917). Serum and EDTA samples were obtained simultaneously during the same venipuncture procedure.

All samples were collected during symptom-free intervals; no blood samples were obtained during acute angioedema attacks.

After blood collection, serum samples were kept at room temperature for approximately 1.5 hours to allow clot formation. Serum was then separated from blood cells by centrifugation according to standardized procedures established in the automated routine clinical laboratory. No deviations from normal laboratory protocols occurred.

Serum ECP levels were measured using the ImmunoCAP^®^ ECP assay (Thermo Fisher Scientific, Waltham, MA, USA), a CE-marked *in-vitro* diagnostic test routinely used in clinical practice. Absolute eosinophil counts were obtained from peripheral blood using standard certified automated hematology analyzers as part of routine complete blood counts.

### Definition of outcomes

As primary outcome, we determined whether ECP levels measured in a real-world setting were specifically increased in cases with confirmed HAE diagnosis. A secondary outcome was the specific increase of absolute eosinophil counts in HAE patients. As exploratory outcomes, we analyzed ECP levels and absolute eosinophil in HAE subgroups (i.e. HAE-C1INH/HAE type 1 and 2, and HAE-nC1INH/HAE type 3) and acquired mast-cell mediated angioedema (denoted as AAE.URT according to the current DANCE classification). Furthermore, we performed sex-stratified analyses on all HAE types and, for balanced comparison, restricted to HAE-C1INH only.

### Statistical analysis

All analyses were conducted in R (version 4.4.3, 2025-02–28 ucrt; R Foundation for Statistical Computing, Vienna, Austria; (URL: http://www.r-project.org/, last accessed 29 Sep 2025). The workflow relied on a modern *tidyverse*-based data structure and visualization ecosystem, and on specialized causal-inference and Bayesian tools. We used tibble (modern data frames), dplyr (data manipulation), tidyr (data reshaping), stringr (string handling), and forcats (factor handling). For visualization we used ggplot2 as the core plotting engine, ggpubr for multi-panel figure assembly, ggnewscale for multiple independent color/fill scales, ggbeeswarm for compact raw-data overlays, ggtext for rich-text axis labels, and scales for axis transformations. Bayesian visualization and posterior summarization used bayesplot, bayestestR, and ggdist. Covariate balance diagnostics were performed using cobalt. Propensity scores were estimated via penalized Firth logistic regression using logistf, ensuring stable estimation under data sparsity. Inverse-probability weighting and post-stratification incorporated Dirichlet draws from *MCMCpack*. Outcome modeling was performed with *INLA* (Integrated Nested Laplace Approximation; URL: http://www.r-inla.org/, last accessed 29 Sep 2025), using spline bases from splines for nonlinear age and seasonality effects. E-values for unmeasured-confounding sensitivity analyses were computed using the EValue package. Word-export and table generation used officer and flextable. File naming was handled using filenamer.

The interaction between data variables in the study was visualized and analyzed using DAG as implemented with *dagitty* (URL: https://www.dagitty.net/dags.html, last accessed 29. Sep.2025). The dagitty code for our DAG is provided in the **Supplementary Materials**.

Power calculations for the Student’s t-test were performed using the *pwr* package’s *pwr.t2n.test* function. The assurance metric, representing the Bayesian probability of study success given the prior uncertainty, was calculated using the package bayesassurance with the function assurance_nd_na (37, 38). A power of 0.8 is generally considered adequate for study planning, whereas no universally accepted target value exists for the assurance metric. The R script for power and assurance calculations (Commented_Bayesian power calculation.R) is provided in the **Supplementary Materials**.

For data analysis, we used a multilevel regression and poststratification (MRP) framework (39). To obtain unbiased and efficient estimates of HAE’s effect on ECP levels and eosinophil counts, we implemented a doubly robust causal-inference strategy that incorporated adjustment variables both in the propensity score model and in the outcome regression (40, 41). For regression analyses, especially those requiring hierarchical model formulations, we used Integrated Nested Laplace Approximation (INLA), a Bayesian inference framework developed by Rue, Martino, and Chopin (42).

In this framework, the variable traditionally referred to as treatment in causal inference $T_{i}$, represents HAE status (1 = HAE case, 0 = non-HAE control). Although HAE status is not a treatment in the clinical sense, we retain the standard causal-inference terminology –specifically the ATT (Average Treatment Effect on the Treated) and ATO (Average Treatment Effect for the Overlap Population) estimands – because these definitions refer to the target population of the estimand rather than to an actual therapeutic intervention. This convention is widely used in observational causal inference for exposures rather than treatments.

Propensity scores were estimated using penalized Firth logistic regression, predicting each observation’s probability of belonging to the HAE cohort given the adjustment variables (41, 43). Firth regularization was chosen instead of standard logistic regression to prevent bias and instability arising from data sparsity or separation (44).

Inverse-probability treatment weights (IPTW) were derived for the ATT estimand and, in sensitivity analyses, for the ATO estimand. Let $p_{i}=P(T_{i}=1|X_{i})$ denote the propensity score of patient with *PatientID* i. We defined the weights as:

ATT weights: $w_{i}^{ATT}=\{\begin{array}{c} 1,\;T_{i}=1\\ \frac{p_{i}}{1-p_{i}}\;,\;T_{i}=0 \end{array}$

ATO weights: $w_{i}^{ATO}=\{\begin{array}{c} 1-p_{i},T_{i}=1\\ p_{i},\;T_{i}=0 \end{array}$

The resulting effective sample size (ESS) was calculated to quantify the amount of information retained after inverse-probability weighting. For a set of weights $w_{i}$, the ESS is defined as


$$\text{ESS}=\frac{{\left(\sum_{i}w_{i}\right)}^{2}}{\sum_{i}w_{i}^{2}},$$

which corresponds to the size of an equivalent sample with equal weights that would yield the same level of precision (45). ESS was computed separately for the HAE group and the control group for both the ATT and ATO weighting schemes.

The IPTW approach is well suited for repeated-measures data because weights operate at the level of individual observations, allowing multiple measurements per patient to be incorporated without compromising the causal interpretation. In INLA, inverse-probability weights were supplied through the weights argument, which reweights each observation’s contribution to the likelihood and thus implements standard IPTW in a Bayesian framework. To account for within-subject correlation, the outcome model included a patient-specific random intercept using f(PatientID, model=“iid”), capturing individual-level heterogeneity across repeated measurements.

To mitigate selection bias due to non-random measurement times, we incorporated the weekday of blood collection as a bias-breaking variable, following the approach of Geneletti et al. within our multilevel MRP framework (46, 47). In detail, the Geneletti method applies a bias breaking variable B that fulfils the following basic independence assumption:


HAE status⊥ Selection into cohorts | ECP value, B

Using DAG analysis, we determined the weekday of blood collection as an optimal bias breaking variable, because it does not influence the ECP value, but has a considerable impact on the selection of patients into the cohort.

The Geneletti method is described for odds ratios, while our log ECP value y is a continuous variable. Therefore, we need to adapt the calculation. For each weekday, we determine the difference in log ECP values using our linear regression model described above.


$$Y_{i}\;∼\;N(\mu_{i},\sigma ),\;\mu_{i}=(\beta_{0}+\gamma_{B_{i}})+(\beta_{HAE}+\delta_{B_{i}})X_{i}+\ldots \;$$

with the stratum (weekday) specific HAE effect ${\text{Δ}}_{b}$ being


$${\text{Δ}}_{b}=\beta_{HAE}+\delta_{b}$$

We are interested in an debiased effect estimator Δ, which can be calculated given weekday probabilities p(B=b), i.e. the probability that a patient was included on a specific weekday. We can calculate this by marginalizing over all K weekdays


$$\text{Δ}=\;\sum_{b}{\text{Δ}}_{b}\cdot p(B=b)\;,\;b\;\in 1,\ldots ,K$$

However, while the effect estimates for ${\text{Δ}}_{b}$ are the posterior distributions inferred from the INLA regression, the weekday probabilities p(B=b) are in fact unknown in our case. We addressed this by resampling both the weekday-specific effect estimates and the weekday probabilities. The latter were drawn from a Dirichlet distribution with varying dispersion parameter κ, representing different assumptions about the degree of uncertainty in weekday selection:


$${\text{Δ}}_{b}^{\left(s\right)}\sim \pi ({\text{Δ}}_{b}|D)$$

$$p{\left(B\right)}^{\left(s\right)}\sim \;Dirichlet(\alpha )\;,\;\;\alpha =\kappa \cdot 1_{K}$$

$${\text{Δ}}^{\left(s\right)}=\;\sum_{b}{\text{Δ}}_{b}^{\left(s\right)}\cdot p{\left(B=b\right)}^{\left(s\right)}$$

We set κ=0.5 (minimally informative Dirichlet prior/Jeffrey’s prior) for *high dispersion*, κ = 10 (medium dispersion) and κ=20 (low dispersion).

By aggregating samples values ${\text{Δ}}^{\left(s\right)}$ obtained above, we approximated the posterior distribution of the overall effect estimator  Δ:


$$\text{p}(\text{Δ|D})\approx \frac{1}{S}\;\sum_{s=1}^{S}\delta_{{\text{Δ}}^{\left(s\right)}}(\text{Δ}),$$

where $\delta_{{\text{Δ}}^{\left(s\right)}}(\cdot )$ denotes the Dirac measure, i.e. a unit mass centered at ${\text{Δ}}^{\left(s\right)}$, representing one posterior draw.

Using INLA, we discerned posterior distributions of model parameters, with the *bayestestR* R package aiding in the extraction of the maximum a posteriori estimator, 95% highest posterior density interval, and Bayesian p-value. This Bayesian p-value aligns closely with its frequentist equivalent, although it differs conceptually, as it reflects the probability that the parameter lies above or below zero given the data (48). We therefore considered a Bayesian p-value ≤ 0.05 as *statistically significant*.

To give an estimate of the robustness of our estimates, we further calculated the Evalues by VanderWeele et al. (49).

### Data and code availability statement

Due to the rare-disease context, the small cohort size, and the longitudinal structure of the data, patient-level raw data cannot be fully anonymized without loss of essential information and are therefore not made publicly available in accordance with GDPR requirements.

Fully commented R scripts, model specifications, directed acyclic graph (DAG) code, and aggregated summary data supporting the findings of this study are provided in the **Supplementary Materials**. De-identified patient-level data may be made available from the corresponding author upon reasonable request and subject to appropriate ethical and data protection approvals.

## Results

### Data collection

The final analysis included 1,928 patients who visited the allergy outpatient clinic of our department and had serum ECP levels measured (ECP measurement cohort, **Table 1**). Absolute eosinophil counts were available for 1,119 of these patients (eosinophil measurement cohort). The cohort comprised approximately twice as many female as male patients, with a median age of 45–47 years. Among all patients, 48 had a confirmed diagnosis of hereditary angioedema (HAE): 32 with HAE-C1-INH type I, none with type II, and 16 with HAE with normal C1-INH (HAE-nC1INH). Patients contributed variable numbers of repeated measurements for both ECP and eosinophil counts. On average, HAE patients had 2.71 ECP measurements compared to 1.25 in non-HAE controls, with similar patterns in eosinophil count measurements.

**Table 1:** Demographic and clinical characteristics of patient cohorts.

Characteristic		ECP measurement cohort		Eosinophil measurement cohort	
		HAE^1^	Non-HAE^1^	HAE^1^	Non-HAE^1^
Demographics	Patients (N)	48	1880	47	1072
	Age (years, mean ± SD)	42.1 ± 17.6	45.8 ± 16.7	42.4 ± 17.6	46.2 ± 16.2
	Age (years, median [IQR])	46.2 [26.8; 54.0]	45.0 [32.4; 58.0]	46.5 [27.5; 54.0]	47.0 [34.0; 58.0]
	Sex: male	17 (35.4%)	528 (28.1%)	16 (34.0%)	268 (25.0%)
	Sex: female	31 (64.6%)	1352 (71.9%)	31 (66.0%)	804 (75.0%)
AAE/HAE type	HAE-C1INH (HAE1/2)	32	0	31	0
	HAE-nC1INH (HAE3)	16	0	16	0
	Mast-cell mediated angioedema (AAE-URT)	6	286	6	41
Comorbidities (ICD10–3 letter codes)	C96 – Other and unspecified malignant neoplasms of lymphoid, hematopoietic and related tissue	0 (0.0%)	13 (0.7%)	0 (0.0%)	10 (0.9%)
	D47 – Other neoplasms of uncertain or unknown behavior of lymphoid, hematopoietic and related tissue	2 (4.2%)	118 (6.3%)	2 (4.3%)	98 (9.1%)
	D89 – Other disorders involving the immune mechanism, not elsewhere classified	2 (4.2%)	81 (4.3%)	2 (4.3%)	62 (5.8%)
	E85 – Amyloidosis/Autoinflammatory disease	0 (0.0%)	38 (2.0%)	0 (0.0%)	37 (3.5%)
	G93 – Other disorders of brain/ME/CFS	0 (0.0%)	22 (1.2%)	0 (0.0%)	19 (1.8%)
	H10 – Conjunctivitis	0 (0.0%)	49 (2.6%)	0 (0.0%)	14 (1.3%)
	J30 – Vasomotor and allergic rhinitis	6 (12.5%)	570 (30.3%)	5 (10.6%)	280 (26.1%)
	J32 – Chronic sinusitis	0 (0.0%)	32 (1.7%)	0 (0.0%)	31 (2.9%)
	J33 – Nasal polyp	0 (0.0%)	16 (0.9%)	0 (0.0%)	14 (1.3%)
	J45 – Asthma	0 (0.0%)	90 (4.8%)	0 (0.0%)	50 (4.7%)
	L20 – Atopic dermatitis	1 (2.1%)	133 (7.1%)	1 (2.1%)	68 (6.3%)
	L23 – Allergic contact dermatitis	0 (0.0%)	94 (5.0%)	0 (0.0%)	41 (3.8%)
	L27 – Dermatitis due to substances taken internally	0 (0.0%)	12 (0.6%)	0 (0.0%)	4 (0.4%)
	L29 – Pruritus	0 (0.0%)	18 (1.0%)	0 (0.0%)	14 (1.3%)
	L30 – Other dermatitis	2 (4.2%)	40 (2.1%)	2 (4.3%)	23 (2.1%)
	L50 – Urticaria	6 (12.5%)	489 (26.0%)	6 (12.8%)	390 (36.4%)
	T63 – Toxic effect of contact with venomous animals	0 (0.0%)	89 (4.7%)	0 (0.0%)	30 (2.8%)
	T78 – Adverse effects, not elsewhere classified	6 (12.5%)	750 (39.9%)	6 (12.8%)	468 (43.7%)
	Z88 – Personal history of allergy to drugs, medicaments and biological substances	5 (10.4%)	258 (13.7%)	5 (10.6%)	122 (11.4%)
Lab. values	ECP (N)	130	2349	124	1386
	Avg. ECP measurements per patient	2.71	1.25	2.64	1.29
	ECP^2^ (median [IQR][min;max])	32.1 [21.0; 48.6] [5.5; 189.0]	21.7 [13.5; 35.8] [2.1; 191.0]	31.8 [20.9; 46.1] [5.5; 189.0]	21.9 [13.4; 37.0] [2.1; 191.0]
	Absolute Eosinophil counts (N)			124	1386
	Avg. Eosinophil counts measurements per patient			2.64	1.29
	Eosinophils absolute^2^ (median [IQR][min;max])			0.14 [0.09; 0.24] [0.00; 0.88]	0.12 [0.07; 0.24] [0.00; 1.70]
	Eosinophils percent^2^ (median [IQR][min;max])			1.90 [1.30; 3.12] [0.0; 10.6]	1.90 [1.10; 3.40] [0.0; 20.9]

^1^If not indicated otherwise: Number (N) of patients with the respective characteristic, and percentage of total (%).

^2^Reference ranges were 0 - 13.3 µg/L for serum ECP, 0.02 - 0.75/nL for absolute eosinophil counts, and 0.5 -5.5% for relative eosinophil counts.

For transparency, cohorts were additionally stratified by sex, and sex-specific demographic characteristics and measurement frequencies are provided in **Supplementary Table 1**. Sex-stratified analyses were performed to assess the robustness of the main findings across demographic strata, rather than to formally test sex as an *a priori* effect modifier. Importantly, the cohort of patients with HAE-nC1INH consisted almost exclusively of females (N = 15), with only a single male patient (N = 1). In contrast, patients with HAE-C1INH showed a balanced sex distribution, comprising 16 males and 16 females.

To estimate the strength and representativity of our study, we used both the classical power calculation and the Bayesian assurance value, i.e. the probability of success under varying preconditions (**Supplementary Figure 1**). These calculations were based upon N_HAE_=48 HAE patients and a three times higher number of controls (N_controls_=144), because we intended to increase the power by leveraging the large number of controls. For a one-sided hypothesis (increased ECP values in HAE patients) we found that an increase of about 1.35, equivalent to an effect size (Cohen’s *d*) of 0.5 was sufficient to reach a power of 0.8, and, depending on the a-priori assumed uncertainty in the effect estimator (denoted as τ_Design_) a Bayesian assurance metric of about 0.6 – 0.9, both values indicating a reasonable study design. It has to be noted here that Bayesian analyses do not depend on power calculations, such as frequentist analyses. The assurance value is generally lower than the power value, because it integrates more sources of uncertainty in pre-study assumptions.

For all patients treated in our department, treatment diagnoses were recorded using the ICD10 code for medical billing purposes. From our database, we identified diagnoses known to be associated with type 2 inflammation as potential confounders. These included allergic rhinitis, sinusitis, atopic dermatitis, bronchial asthma, chronic spontaneous mast cell-mediated angioedema, and chronic spontaneous urticaria. In cases where patients had multiple ECP measurements and potentially different diagnosis codes associated with each measurement, we combined all recorded diagnosis codes. It is important to note that all the mentioned diagnoses are chronic conditions. We assumed that differences in the recorded diagnosis codes were more likely due to documentation errors rather than the presence or absence of the respective disease.

Considering the substantial number of patients with allergic rhinitis in our database, we included the month of blood collection as a seasonal factor. Particularly, for pollen allergies, we anticipated an increase in eosinophilic inflammation during spring and summer.

### Bias-breaking via multilevel regression and poststratification

Our retrospective study design is inherently prone to selection bias. Although our outpatient clinic is integrated into a general allergy service, it has a particular focus on hereditary angioedema, and patients with the full spectrum of allergic, inflammatory, and allergy-like diseases are seen during the same consultation hours. Patient recruitment and sample collection varied across weekdays, as different physicians were responsible for each clinic day. Consequently, inclusion into the analytic cohort is moderately dependent on the weekday. In contrast, the clinical laboratory follows a standardized and largely automated workflow throughout the week, ensuring that ECP measurements and complete blood counts can be considered independent of weekday-related factors.

**Figure 1:**
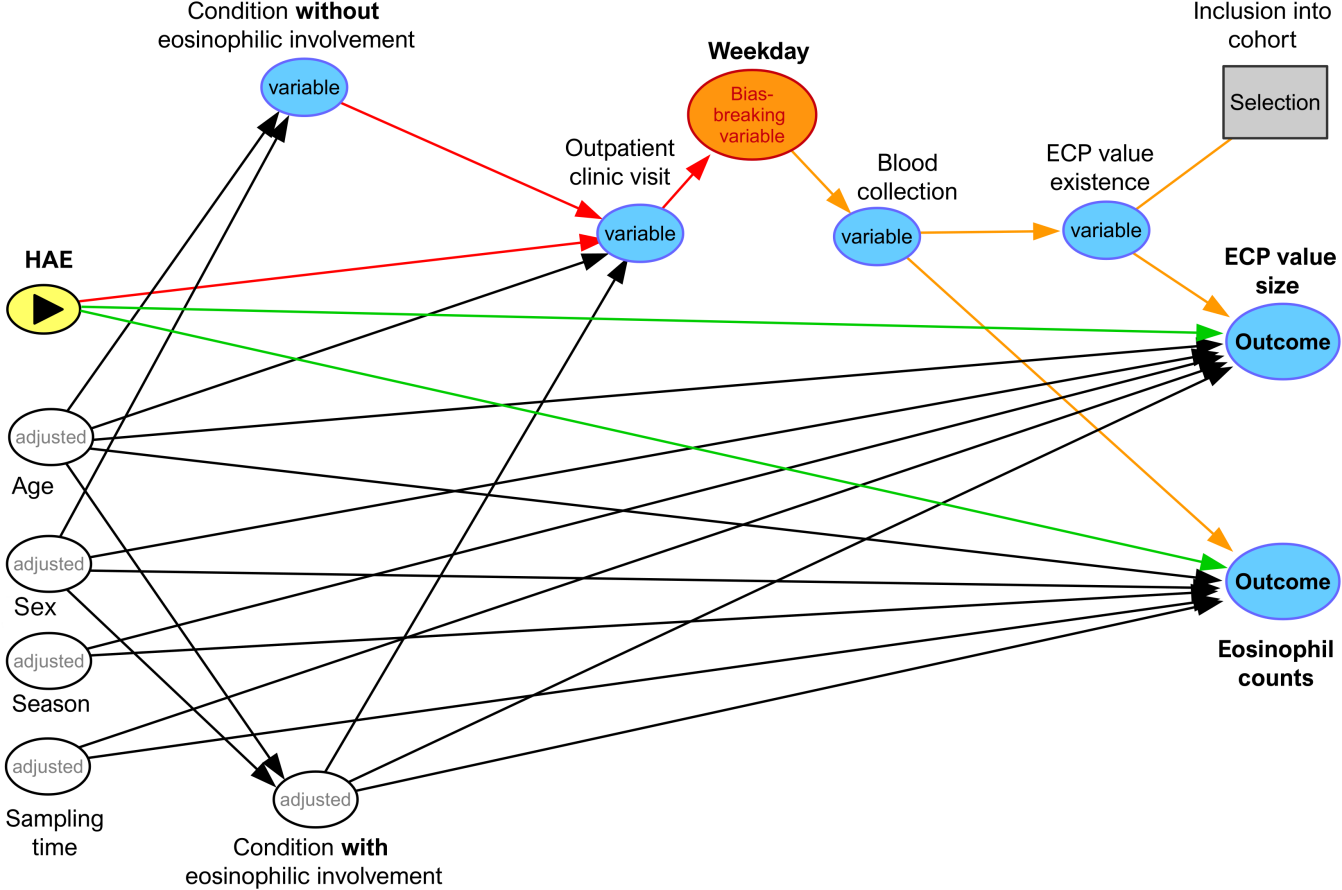
Directed acyclic graph representing assumed causal relations between exposure, covariates, measurement process, and outcomes. The DAG was constructed, analyzed, and exported using *dagitty* (https://www.dagitty.net/dags.html; last accessed September 15, 2025) and OpenOffice Draw. Green arrows indicate the putative causal effects of hereditary angioedema (HAE) on the outcomes serum eosinophil cationic protein (ECP) levels and eosinophil counts. Red arrows denote biasing paths (backdoor or selection-induced), while orange arrows highlight biasing paths that are blocked by a designated bias-breaking variable. Diagnoses coded at the three-letter ICD-10 level were grouped into conditions with eosinophilic involvement - expected to influence ECP levels and eosinophil counts - and conditions without eosinophilic involvement. Together with age, sex, sampling time, and season (month), conditions with eosinophilic involvement constituted the prespecified adjustment set (used both for regression adjustment and inverse probability treatment weighting). Inclusion in the cohort required availability of an ECP measurement, which depends on the care and measurement process (outpatient visit scheduling → weekday → blood collection) as well as the database query. This conditioning opens a selection path via cohort inclusion. To mitigate this source of selection bias, the weekday of blood collection was treated as a bias-breaking variable: weekday is associated with clinic scheduling and thus selection (e.g., disease-specific outpatient clinics held on particular weekdays) but is not expected to causally affect ECP levels or eosinophil counts, as laboratory procedures are constant across weekdays. Although adjustment for age, sex, season (month), sampling time, and eosinophil-related comorbidities reduces confounding, unmeasured confounding may persist (not represented in the graph). Therefore, E-values were additionally computed (VanderWeele) to quantify the minimum strength of unmeasured confounding required to fully explain away the observed associations.

We used dagitty to visualize and analyze the assumptions about the interactions of data variables in a directed acyclic graph (DAG) (**Figure 1**). The HAE status, which was additionally curated in the database, is the exposure, and we are interested in its causal effect on the ECP level and eosinophil counts (absolute) as outcomes. The existence of the ECP value is the precondition for inclusion into the cohort (selection variable). Age, sex, season of the year, (recorded) sampling time and comorbidities that have a potential influence were used as adjustment variables, while comorbidities such as type IV sensitization against contact allergens without present disease are considered as having no influence on the ECP value, but lead to presentation in the outpatient clinic with ECP value determination and therefore selection into the cohort.

Using *dagitty*, causal and biasing paths can be identified by specifying the attributes of the nodes. In our DAG, “inclusion into the cohort” acts as a collider, while the biasing path runs through the weekday variable. Accordingly, poststratification can block this biasing path and thus enable estimation of an unbiased causal effect of HAE status on ECP levels and eosinophil counts. Age, sex, season, and eosinophil-associated conditions were treated as confounders and controlled for through doubly robust regression, further reducing potential bias. Although not depicted in the DAG, unmeasured confounding could still introduce bias; to assess the robustness of our findings, we therefore calculated E-values according to VanderWeele et al. (49). These considerations justify the use of weekday as a bias-breaking variable.

The original Geneletti approach for bias-breaking variables relies on external reference data for poststratification. Because such external weekday distributions were unavailable, we instead implemented Bayesian bootstrapping, assuming a variable distribution of weekday frequencies modeled by a Dirichlet distribution with equal base parameters but varying dispersion (low, moderate, and high). This dispersion propagates into the final posterior estimate of the HAE effect as an additional layer of uncertainty, mathematically attenuating statistical significance and thereby yielding highly conservative effect estimates.

### ECP levels are specifically increased in hereditary angioedema

**Figure 2:**
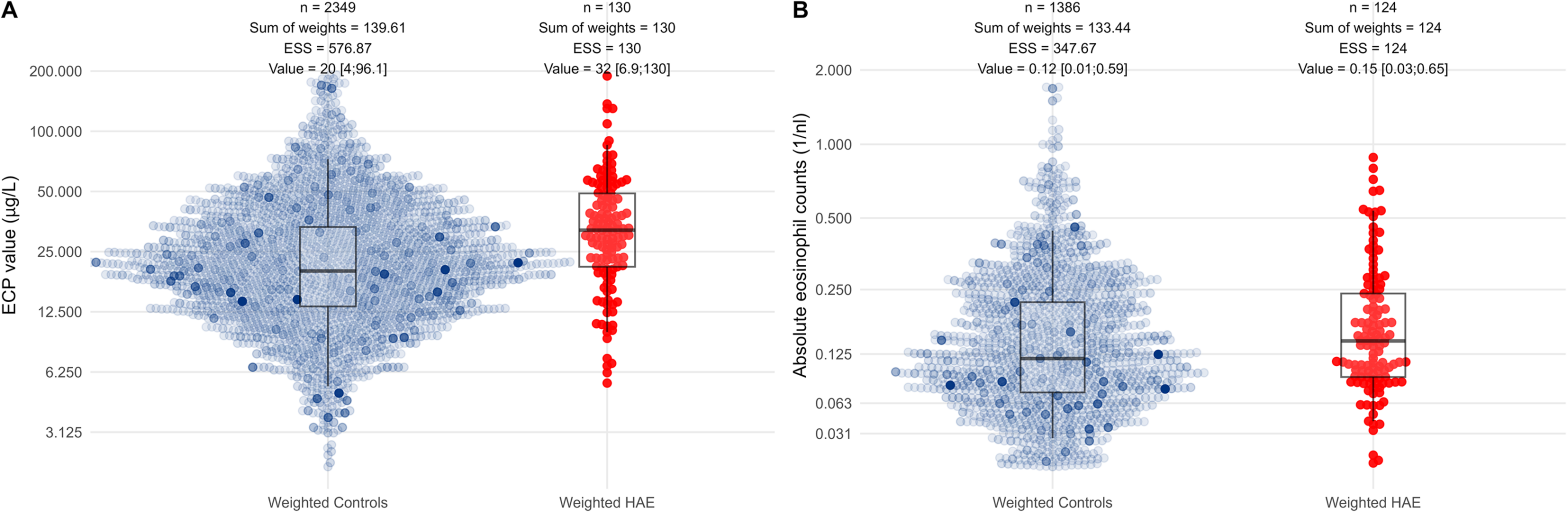
Visualization of raw ECP and eosinophil count data. Combined box-and-beeswarm plots of **(A)** eosinophilic cationic protein (ECP) levels and **(B)** absolute eosinophil counts. Each dot represents an individual measurement from a patient with hereditary angioedema (HAE, red) or from a non-HAE control (blue-grey). Dot opacity reflects ATT propensity-score weights applied to the control group: more opaque points carry higher weights. For visualization, scales for ECP concentrations were log-transformed [ log(x)], and scales for eosinophil counts were transformed using the inverse hyperbolic sine [ asinh(x / c)]; both transformations (including the choice of c) were selected to approximate normality. A small random jitter was added to mitigate discretization. Median values, interquartile ranges, whisker limits, the number of measurements, the sum of ATT weights, the effective sample size (ESS), and the weighted median and 0.025 – 0.975 quantiles are shown above each group; all summary statistics are based on weighted quantiles, meaning the boxplots reflect the ATT weights. Boxplots indicate higher ECP levels in HAE patients compared with weighted controls, whereas eosinophil counts show only minimal differences between groups.

The primary outcome was the serum ECP level. We tested whether patients with hereditary angioedema (HAE) exhibited higher ECP concentrations compared to non-HAE controls (**Figure 2**). All available ECP measurements from the 48 HAE patients and all controls were included. Propensity scores were estimated using Firth logistic regression based on covariates identified in the directed acyclic graph (sex, age, comorbidities, and month of sample collection). These covariates were used both for constructing inverse-probability-of-treatment weights (ATT estimand) and, in the doubly robust models, as covariates in the outcome regression. The weekday of blood collection was incorporated as a bias-breaking variable based on the Geneletti criterion.

Among 2,349 control measurements, ATT weighting yielded an ESS of 576.87 and a weighted median ECP level of 20 µg/L. Among 130 HAE measurements, the ESS equaled the observed sample size (130), with a weighted median of 32 µg/L (**Figure 2**). Because repeated measurements inflate simple descriptive statistics, direct group comparisons are not appropriate; therefore, we used hierarchical linear models with patient-specific random intercepts to accommodate multiple measurements per patient.

**Figure 3:**
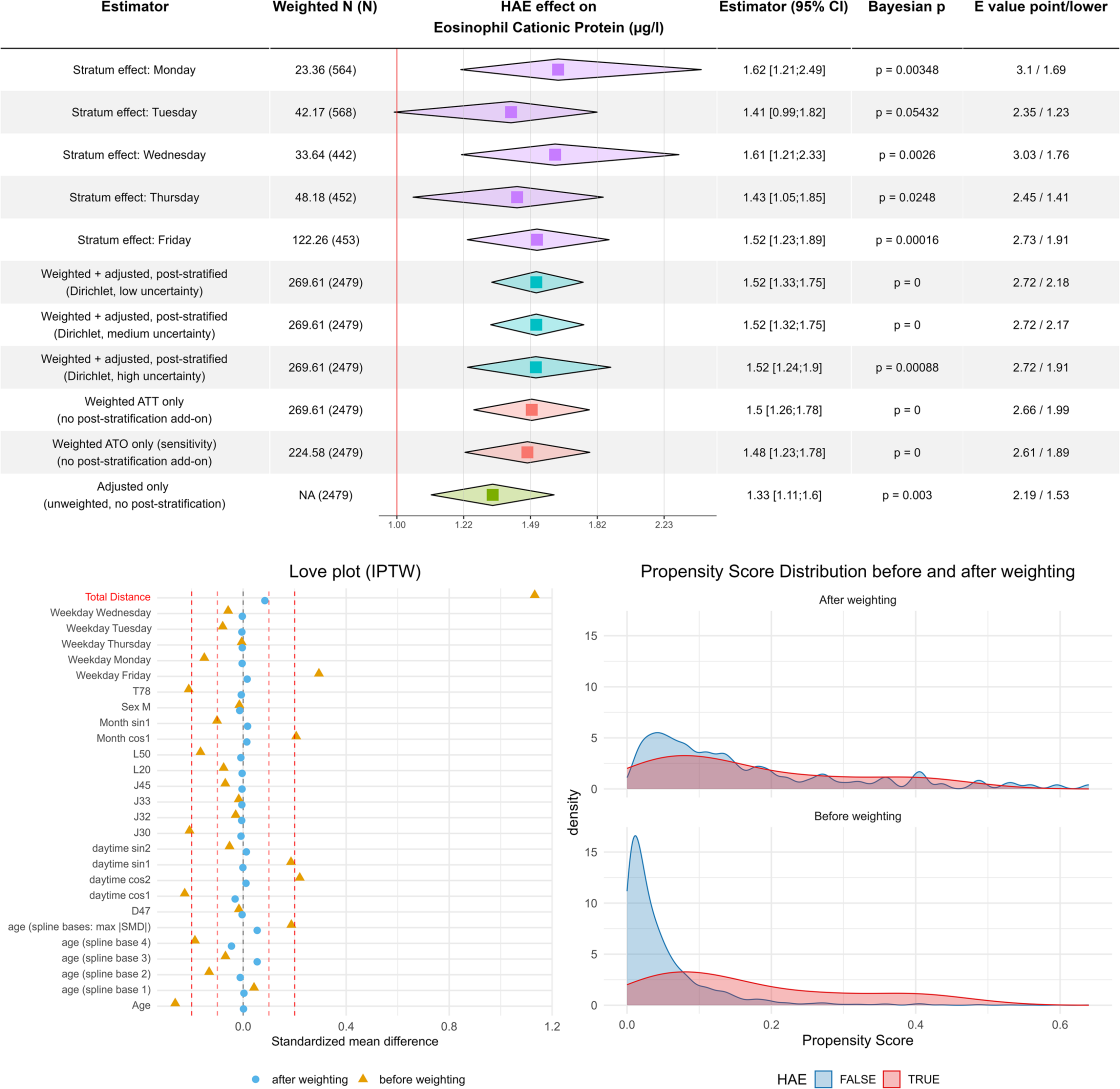
Eosinophilic cationic protein levels are increased in hereditary angioedema. Combined tabular–forest plot summarizing estimated effect sizes for the increase in serum log-transformed [ log(x)] ECP levels associated with hereditary angioedema (HAE) across multiple model specifications addressing the same causal contrast. Rows 1–5 display weekday-specific stratum effects from a hierarchical INLA regression model. Rows 6–8 present ATT-weighted, covariate-adjusted, and Dirichlet post-stratified (“doubly robust”) estimates, using Dirichlet weights with low (row 6), medium (row 7), and high uncertainty (row 8) in weekday frequencies. Rows 9–11 show singly robust models, applying ATT weighting alone (row 9), ATO weighting for sensitivity analysis alone (row 10), and covariate adjustment alone (row 11), each without post-stratification. Squares represent posterior medians; the lateral corners of each diamond represent the 2.5% and 97.5% quantiles of the posterior effect distribution. Bayesian p-values (probability of direction) and VanderWeele E-values for both the median and the lower (2.5%) quantile quantify evidence strength and sensitivity to unmeasured confounding. The lower-left panel (Love plot) displays standardized mean differences before and after propensity-score–based ATT weighting, demonstrating excellent covariate balance (standardized mean difference ≈0.1). The lower-right panel shows kernel-density estimates of the propensity-score distributions before and after ATT weighting, confirming substantial overlap between HAE and control groups.

In contrast to conventional matching approaches, we used inverse-probability-of-treatment weighting (IPTW) to retain all observations and avoid unnecessary data loss. The resulting weights achieved satisfactory covariate balance and adequate overlap between HAE and control patients. Propensity score distributions after weighting are shown in **Figure 3**, demonstrating substantial common support for estimating the ATT. Covariate-wise standardized mean differences (SMDs) are presented in the corresponding Love plot (**Figure 3**), confirming that all covariates reached acceptable balance after weighting, i.e. a standardized mean difference of below 0.1.

Rather than reporting a single adjusted estimate, we constructed a set of complementary regression specifications, each targeting the same causal contrast (the effect of HAE on ECP levels) under different statistical assumptions (**Figure 3**). These included (1): a naïve unweighted regression (2); IPTW-only models (3); doubly robust models (IPTW with covariate adjustment) (4); Bayesian poststratification using low-, medium-, and high-dispersion Dirichlet priors to reflect increasing uncertainty in the weekday distribution; and (5) an analysis using overlap (ATO) instead of ATT weights. Weekday-specific HAE effects were additionally summarized in the result table. Together, these models constitute a structured series of sensitivity analyses that assess the stability of the estimated effect across a wide analytical space.

Among these specifications, the post stratified model with high Dirichlet dispersion – which assumes the greatest uncertainty regarding weekday sampling – yields the most conservative estimate and was therefore designated as the primary effect measure. Even under this highly conservative scenario, HAE was associated with a 1.52-fold increase in ECP levels (95% credibility interval: 1.24–1.90; Bayesian p = 0.00088). This corresponds to a moderate effect size consistent with biologically plausible differences in eosinophil activation. The VanderWeele E-value was 2.72 (1.91 under the most conservative assumptions), indicating substantial robustness to unmeasured confounding. Of note, the ATT estimator yields a larger effect size than the unweighted estimator because it directly targets the counterfactual question of how ECP levels would differ within the subgroup of HAE patients if these patients did not have HAE. By reweighting control observations to resemble the covariate distribution of HAE patients, the ATT focuses on the clinically relevant comparison for this population.

Because our cohort included a relatively large number of patients with HAE-nC1INH (or HAE type 3), we performed additional analyses stratified by HAE subtype: HAE-C1INH (HAE type 1/2, **Supplementary Figure 2**), HAE-nC1INH (HAE type 3, **Supplementary Figure 3**), and mast-cell-mediated angioedema (AAE.URT; **Supplementary Figure 4**). The raw data for each subgroup is shown in **Supplementary Figure 5**. The pattern observed in the overall cohort was reproduced in all subgroups. ECP levels were significantly elevated in HAE-C1INH (1.37-fold increase, p=0.01684) and even more pronounced in HAE-nC1INH (1.7-fold increase, p=0.04268). A small but detectable increase was also observed in mast-cell-mediated angioedema (1.15-fold increase, p=0.01712). However, covariate balance in the subgroup analyses was somewhat reduced compared with the main analysis, with standardized mean differences of up to approximately 0.2 for several covariates in each subgroup. This reflects the smaller sample sizes of the individual strata and should be taken into account when interpreting subgroup results. These findings support the internal validity of our results: HAE-nC1INH patients display a similar and even stronger pattern of eosinophil activation than HAE-C1INH patients, whereas the effect in mast-cell–mediated angioedema is minimal, consistent with the pathophysiology of that condition. Notably, the observation in HAE-C1INH – where the diagnosis can be established with high certainty based on C1-INH functional assays – further supports that the ECP elevation is genuinely related to bradykinin-mediated angioedema rather than misclassification with mast-cell-mediated disorders.

We additionally explored potential sex-specific differences in the association between HAE and ECP levels. Owing to the limited number of male HAE patients and controls, these analyses were exploratory. An elevated ECP level associated with HAE was observed in females (1.6-fold increase, p=0.003, **Supplementary Figure 6**), whereas estimates in males were less precise and did not reach statistical significance (1.28-fold increase, p=0.17308, **Supplementary Figure 7**). Inspection of the raw data suggested slightly higher baseline ECP levels in male compared with female controls, while ECP levels were similar between males and females among HAE patients (**Supplementary Figures 8A, B**).

Notably, inverse probability weighting achieved less satisfactory covariate balance in males, as reflected by standardized mean differences exceeding 0.2, which limits the interpretability of male-specific estimates. Because HAE-nC1INH occurred almost exclusively in females, we performed additional sex-stratified analyses restricted to patients with HAE-C1INH only. In this more balanced subgroup, the association between HAE and elevated ECP levels was directionally consistent in both females (1.44-fold increase, p=0.01324, **Supplementary Figure 9**) and males (1.19-fold increase, p=0.31128, **Supplementary Figure 10**). Corresponding raw data are shown in **Supplementary Figure 11A, B**. Taken together, these findings demonstrate that elevated ECP levels in HAE patients are reproducible across all modeling frameworks and remain robust to increasingly conservative assumptions. The consistency of results across multiple analytic perspectives supports both the stability and the credibility of the estimated HAE effect.

### Eosinophil granulocyte counts are not influenced by HAE

Although ECP levels reflect a different biological aspect of eosinophil activity than absolute eosinophil counts, we examined whether eosinophil counts showed a similar increase in HAE patients (**Figure 2**). The database was primarily designed to extract ECP values from the clinical information system and to include eosinophil counts only when available. Consequently, several eosinophil count values were missing, and one HAE patient lacked eosinophil data (N = 47, with 124 measurements, ESS = 124 with ATT weighting). The number of controls was also smaller (N = 1072, with 1386 measurements, ESS = 347.67 with ATT weighting). The weighted median of absolute eosinophil counts was 0.12/nL in controls and 0.15/nL in HAE cases.

**Figure 4:**
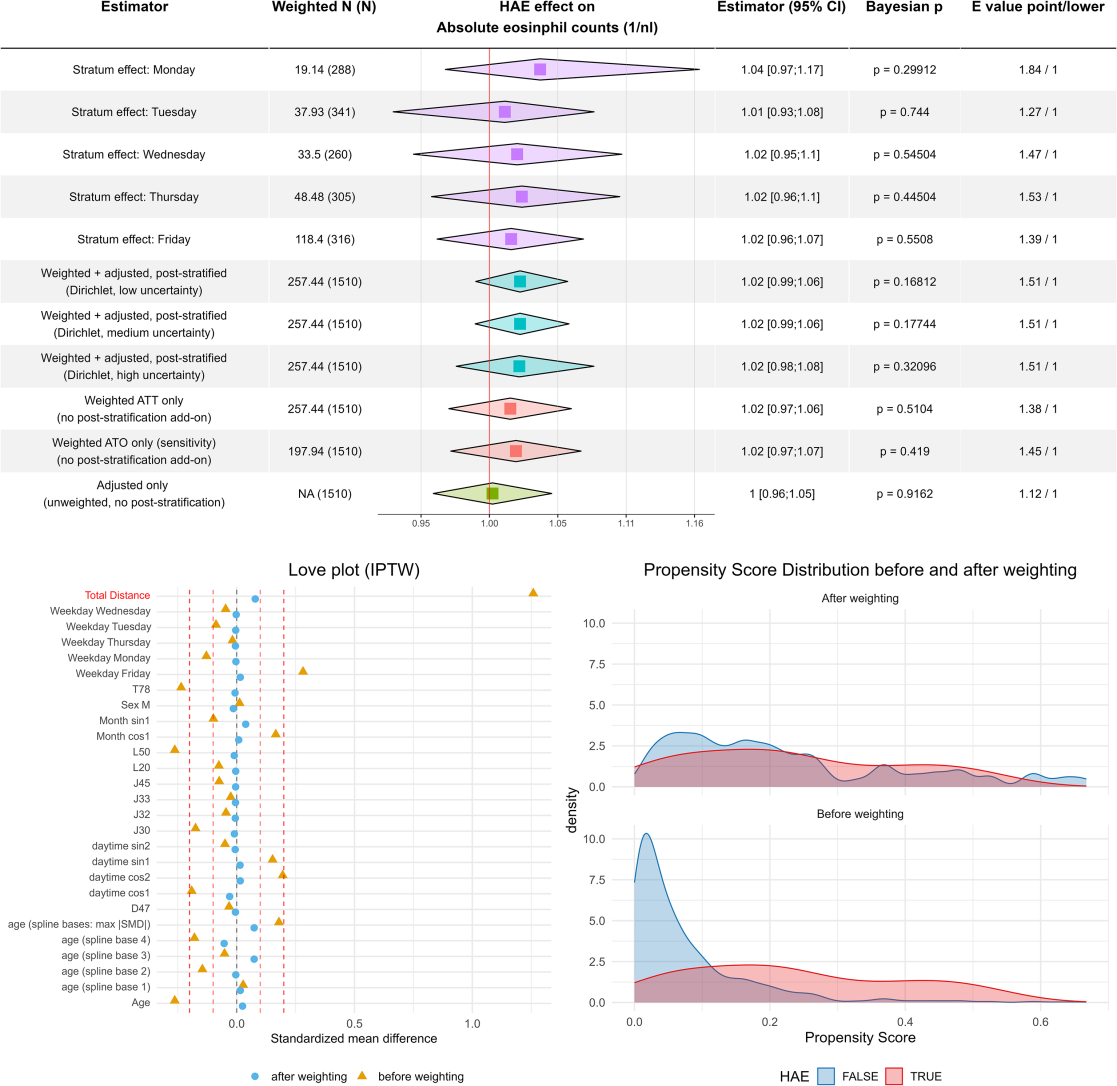
Cell counts of eosinophil granulocytes are not increased in hereditary angioedema. Combined tabular–forest plot summarizing estimated effects of hereditary angioedema (HAE) on absolute eosinophil counts. The modeling framework mirrors the approach used for ECP: Rows 1–5 show weekday-specific stratum estimates derived from the hierarchical INLA model (the Wednesday deviation reflects sparse data and shrinkage toward the prior). Rows 6–8 report doubly robust estimates obtained by combining ATT-IPTW, covariate adjustment, and Dirichlet post-stratification with increasing uncertainty in weekday frequencies. Rows 9–11 display the corresponding singly robust analyses (ATT weighting only, ATO weighting only, and covariate adjustment only), all without post-stratification. Squares denote posterior medians; diamonds indicate 2.5% and 97.5% quantiles. Bayesian p-values (probability of direction) and VanderWeele E-values quantify evidence strength and sensitivity to unmeasured confounding. As in the ECP figure, the lower-left panel shows covariate balance before and after ATT-IPTW (Love plot), and the lower-right panel illustrates propensity-score distributions pre- and post-weighting, indicating maintained overlap between HAE and control groups.

We performed a regression analysis with the same pattern as with the ECP values before (**Figure 4**). The Love plot demonstrated good balance between HAE case and control measurements, as indicated by a standard mean difference below 0.1 and a satisfactory overlap of propensity score distributions after weighting.

The regression analysis itself indicated that HAE had no measurable effect on eosinophil counts across all model specifications. The slightly increased HAE effect in the Monday stratum further illustrates the utility of the post-stratification approach: it highlights that weekdays affect patient selection, whereas eosinophil counts themselves remain unaffected. A genuine weekday-related effect on eosinophil counts would imply a systematic weekday bias in laboratory measurement quality, which is unlikely given the standardized and automated laboratory procedures.

We performed similar subgroup analyses as for the ECP level here: for HAE-C1INH (HAE type 1/2, **Supplementary Figure 12**), HAE-nC1INH (HAE type 3, **Supplementary Figure 13**), and mast-cell-mediated angioedema (AAE-URT, **Supplementary Figure 14**). The raw data comparison (**Supplementary Figure 15**) showed a slight increase in absolute eosinophil counts in HAE-nC1INH cases, compared to corresponding controls. However, this is not reflected in the regression analyses, which confirmed no effect of HAE-C1INH, HAE-nC1INH, or mast-cell mediated angioedema on absolute eosinophil counts. It has to be noted that the weighting was less optimal in subgroup analyses, compared to the main analysis.

Similar results were obtained in the sex-stratified analyses, which were performed equivalently to the ECP analyses. Neither females with HAE (**Supplementary Figure 16**), nor males with HAE (**Supplementary Figure 17**) showed an increase in absolute eosinophil counts. The corresponding raw data is shown in **Supplementary Figures 8C, D**. Restriction to patients with HAE type 1 and 2 did not reveal any effect of the disease on absolute eosinophil counts in females (**Supplementary Figure 18**) or males (**Supplementary Figure 19**). Likewise, the corresponding raw data is shown in **Supplementary Figures 11C, D**.

## Discussion

In this study, we demonstrate that patients with HAE exhibit significantly increased serum ECP levels, while absolute eosinophil counts remain unchanged. This dissociation between biochemical evidence of eosinophil activation and eosinophil number was consistently observed across HAE subtypes, suggesting enhanced eosinophil activation rather than eosinophilia per se. Importantly, this pattern proved robust across multiple analytical strategies, including stratified analyses, correction for selection effects, and sensitivity analyses addressing potential unmeasured confounding.

Rather than relying on fixed point estimates, stratum weights were modeled as Dirichlet-distributed random variables, intentionally shifting the bias-variance trade-off toward increased variance. By accepting reduced statistical efficiency, this strategy prioritizes bias reduction and supports causal interpretation under explicit modeling assumptions, addressing selection-related distortions inherent to retrospective observational designs.

These findings extend the current pathophysiological view of HAE, which has traditionally focused on bradykinin-mediated increases in vascular permeability as the dominant mechanism underlying acute angioedema attacks (1, 2). Our data indicate that, beyond episodic swelling, HAE is associated with a state of low-grade inflammation characterized by biochemical evidence of eosinophil activation, even during symptom-free intervals.

This interpretation is supported by previous reports of persistent complement activation outside clinically manifest attacks, reinforcing the concept of ongoing subclinical disease activity in HAE (29). In line with this framework, recent studies describing activation of inflammatory pathways in HAE further suggest involvement of eosinophil-associated immune mechanisms beyond the acute attack phase (17, 28).

When interpreting the dissociation between elevated ECP levels and unchanged eosinophil counts, it is important to recognize the limited ability of peripheral blood eosinophil numbers to reflect eosinophil activity at the tissue level. Eosinophils may rapidly exit the circulation and exert effector functions within tissues, such that blood counts underestimate biologically relevant activation.

Consistent with this concept, a U-shaped relationship between eosinophil counts and eosinophil-associated organ manifestations has been described, with increased clinical risk observed even at low or normal eosinophil numbers (50). These observations align well with our finding of elevated ECP levels despite normal eosinophil counts and argue against enhanced eosinophil proliferation as the primary mechanism. Instead, our data are more compatible with activation and degranulation of terminally differentiated eosinophils, a dissociation that has also been reported in other inflammatory and vascular conditions.

While our findings support ECP as a marker of eosinophil-related activation, its cellular origin warrants careful consideration. ECP cannot be regarded as a strictly eosinophil-specific mediator, as several human studies have demonstrated ECP presence and, under certain inflammatory conditions, release by other myeloid cell populations, particularly neutrophils and, to a lesser extent, circulating monocytes. Depending on experimental context, ECP detection in neutrophils has been attributed either to endogenous expression or to uptake and intracellular storage of eosinophil-derived ECP (51–54), and low-level ECP expression has also been described in peripheral blood monocytes with loss upon differentiation into macrophages (53).

Against this background, our results should not be interpreted as evidence for eosinophils as the exclusive cellular source of elevated ECP in HAE. Rather, the central observation—the dissociation between elevated ECP levels and unchanged eosinophil counts—supports a model of cellular activation and degranulation rather than proliferation, irrespective of the precise cellular origin of ECP. While eosinophils likely remain the dominant contributors to circulating ECP in many inflammatory conditions, alternative myeloid sources may contribute under specific circumstances, underscoring the need for cautious interpretation of ECP as a biomarker in complex diseases such as HAE.

While direct mechanistic evidence is lacking, indirect observations provide a biologically plausible framework for interpreting our findings. In other clinical contexts, pharmacologic blockade of the bradykinin B2 receptor has been associated with changes in peripheral eosinophil counts, indicating that the kallikrein–kinin axis can interact with eosinophil biology, although such effects are context-dependent and do not establish a mechanism in HAE (55). Conversely, eosinophil-driven angioedema syndromes such as episodic angioedema with eosinophilia (Gleich’s syndrome) demonstrate the reverse sequence, in which eosinophil activation precedes tissue swelling, and experimental data indicate that eosinophilic inflammation can engage kallikrein–kinin and bradykinin-linked pathways upstream of vascular permeability (24–26, 56). Although distinct from HAE, these conditions illustrate that eosinophilic and bradykinin-mediated pathways can converge on shared vascular endpoints, supporting the concept of bidirectional crosstalk between eosinophil activation and edema formation.

In HAE, where dysregulation of the kallikrein–kinin system represents the primary defect, our data are more consistent with secondary eosinophil activation downstream of vascular or endothelial perturbation. This interpretation is supported by recent omics-based studies identifying activation of inflammatory pathways linked to eosinophil biology in HAE (28).

Importantly, eosinophilic inflammation can phenotypically resemble or modulate vascular swelling responses without sharing the underlying pathophysiology of HAE, as illustrated by pseudo-angioedema observed in drug reaction with eosinophilia and systemic symptoms (DRESS) (57). Accordingly, therapeutic responses to eosinophil-targeted interventions reported in eosinophil-driven angioedema syndromes (58) should not be extrapolated to HAE, in which the kallikrein–bradykinin pathway remains the principal therapeutic target.

The consistent elevation of ECP suggests that eosinophil activation may accompany the well-established bradykinin-mediated vascular pathology of HAE as a chronic or recurrent, low-grade inflammatory process. Such activation may reflect downstream immune consequences of ongoing vascular and tissue stress, potentially driven by subclinical complement activation or cytokine-mediated crosstalk between endothelial cells, mast cells, and eosinophils (23). While the mechanistic basis remains speculative, this link between innate vascular inflammation and type-2–associated immune mediators may help explain persistent inflammatory signals in HAE beyond acute attacks.

The clinical relevance of this eosinophilic activation remains to be determined. Persistent complement consumption under effective long-term kallikrein inhibition and the reported increased prevalence of allergic and autoimmune comorbidities in HAE (59–61) support the concept of ongoing subclinical immune activation. It is conceivable that low-grade eosinophil activation contributes to gastrointestinal symptoms, food-related intolerances, or impaired quality of life reported by some patients with HAE (62–64), although this hypothesis requires targeted investigation.

This study has limitations inherent to its single-center, retrospective design and the incomplete availability of eosinophil count data. Diagnostic classification was based on existing clinical documentation, which may have introduced heterogeneity in diagnostic certainty, particularly among patients with HAE-nC1INH. Missing eosinophil count data and potential under-documentation of allergic comorbidities may have resulted in residual confounding. The retrospective design precludes temporal interpretation of cause and effect; thus, it remains uncertain whether eosinophil activation precedes or follows vascular events in HAE.

As this analysis was conducted in a single tertiary care center with an allergy-focused patient population, generalizability to other settings may be limited. Nevertheless, tertiary centers often reflect real-world referral patterns of rare diseases like HAE, lending external relevance despite local sampling. ECP is a biologically variable biomarker sensitive to pre-analytical conditions such as sample handling and timing, which may further contribute to measurement variability.

All HAE (and acquired angioedema) samples in this study were collected during symptom-free intervals. This reduces biological heterogeneity but does not allow temporal profiling of ECP dynamics or eosinophil activation during acute attacks. However, it does allow us to demonstrate that even during attack-free periods, patients with HAE show measurable eosinophil activation, indicating ongoing biological activity between attacks.

Documentation of long-term prophylactic treatment (e.g., lanadelumab or berotralstat) was not uniformly available across historical records. However, ECP measurements in the HAE cohort were predominantly obtained during routine follow-up visits, frequently under ongoing long-term prophylaxis. As these agents target the kallikrein–bradykinin pathway and are not known to directly suppress eosinophil activation, a major confounding effect on baseline ECP levels appears unlikely. Importantly, systemic corticosteroids—which are known to reduce eosinophil counts and ECP levels—were rarely used in both the HAE and control cohorts, as sampling occurred in an elective outpatient setting and not during acute disease exacerbations. Therefore, corticosteroid-related suppression of eosinophil activation markers is unlikely to explain the observed differences. Nevertheless, incomplete documentation of treatment status represents a limitation inherent to the retrospective design. Finally, although the applied Bayesian MRP framework and E-value analysis strengthen causal inference, residual unmeasured confounding cannot be fully excluded.

In summary, this study identifies a previously underrecognized eosinophilic component in HAE, suggesting that subtle inflammatory processes may accompany the well-established bradykinin-mediated mechanisms of the disease. This finding broadens the clinical perspective on HAE, indicating that its manifestations extend beyond acute, life-threatening attacks and may include low-grade inflammatory activity with potential implications for patient well-being. Although the methodological framework ensured robust estimates, the principal value of this work lies in demonstrating how carefully analyzed routine clinical data can yield meaningful biological insights. While serum ECP levels may serve as a useful population-level indicator of eosinophil activation, they are not suitable for diagnostic application in individual patients. Future studies should aim to validate these findings and to clarify how chronic eosinophilic activation may influence disease expression, comorbidities, and quality of life in HAE.

## Data Availability

Due to the rare-disease context, the small cohort size, and the longitudinal structure of the data, patient-level raw data cannot be fully anonymized without loss of essential information and are therefore not made publicly available in accordance with GDPR requirements. Fully commented R scripts, model specifications, directed acyclic graph (DAG) code, and aggregated summary data supporting the findings of this study are provided in the **Supplementary Material**. De-identified patient-level data may be made available from the corresponding author upon reasonable request and subject to appropriate ethical and data protection approvals.
