# Supplementary material for: Eosinophilic inflammation in hereditary angioedema: a single-center real-world retrospective chart review study
Source: Front Immunol. 2026 Feb 17;17:1754405. doi: 10.3389/fimmu.2026.1754405 (PMC12953394; doi:10.3389/fimmu.2026.1754405)
Supplement: Supplementary file 1 [file DataSheet1.pdf]

Supplementary data overview

Supplementary Files

**Filename**	**Description**
Commented_analysis_public_data_Males_and_Females.R (part of SupplementaryData.zip archive)	R script that analyzes the data sets and produces the main plots, including sex stratified tables and plots
Commented_Bayesian power calculation.R (part of SupplementaryData.zip archive)	R script that performs the power and assurance calculation
DAG.txt (part of SupplementaryData.zip archive)	Dagitty code of the Directed Acyclic Graph of variable interactions in our data set
Supplementary Table Sex-stratified demographics.pdf	Sex-stratified demographics table
Supplementary Figure 1 – PowerAssuranceSupplement.pdf	Supplementary Figure 1
supplementary-figure-2---hae1_ecp_analysis_qc_20260112T222257.tiff	Supplementary Figure 2
supplementary-figure-3---hae3_ecp_analysis_qc_20260112T222318.tiff	Supplementary Figure 3
supplementary-figure-4---aaeurt_ecp_analysis_qc_20260112T222335.tiff	Supplementary Figure 4
supplementary-figure-5---raw_supplement_ecp_qc_20260112T222639.tiff	Supplementary Figure 5
supplementary-figure-6---females_hae_ecp_analysis_qc_20260112T222405.tiff	Supplementary Figure 6
supplementary-figure-7---males_hae_ecp_analysis_qc_20260112T222348.tiff	Supplementary Figure 7
supplementary-figure-8---male_female_raw_plot_qc_20260112T222607.tiff	Supplementary Figure 8
supplementary-figure-9---females_hae12_ecp_analysis_qc_20260112T222433.tiff	Supplementary Figure 9
supplementary-figure-10---males_hae12_ecp_analysis_qc_20260112T222417.tiff	Supplementary Figure 10

supplementary-figure-11---male_female_hae12_raw_plot_qc_20260112T222636.tiff	Supplementary Figure 11
supplementary-figure-12---hae1_eos_analysis_qc_20260112T222506.tiff	Supplementary Figure 12
supplementary-figure-13---hae3_eos_analysis_qc_20260112T222523.tiff	Supplementary Figure 13
supplementary-figure-14---aae_urt_eos_analysis_qc_20260112T222537.tiff	Supplementary Figure 14
supplementary-figure-15--raw_supplement_eosinophils_qc_20260112T222641.tiff	Supplementary Figure 15
supplementary-figure-16---females_hae_eosinophil_analysis_qc_20260112T222553.tiff	Supplementary Figure 16
supplementary-figure-17---males_hae_eosinophil_analysis_qc_20260112T222605.tiff	Supplementary Figure 17
supplementary-figure-18---females_hae12_eosinophil_analysis_qc_20260112T222623.tiff	Supplementary Figure 18
supplementary-figure-19---males_hae12_eosinophil_analysis_qc_20260112T222635.tiff	Supplementary Figure 19

Data specification

The raw data files *Raw_ECP_database.csv* and *Raw_Eos_database.csv* are semicolon-separated and use double quotation marks for string fields.

Both datasets were generated from original clinical information system exports and then processed to fully de-identify all patient information.

Raw_Eos_database.csv is a reduced subset of Raw_ECP_database.csv, containing only those observations for which both ECP measurements and eosinophil counts were available.

**Column name**	**Column data type**	**Description**
Age	Numeric	Age at the time of blood collection (years)
Gender	Character (Factor)	“F” for female, “M” for male
Collection.Month	Integer	Month of sample collection (1–12)
Weekday	Character (Factor)	Weekday name of sample collection

Column name	Column data type	Description
ECP.value	Numeric	Eosinophilic cationic protein (ECP) in µg/L
logECP[1]	Numeric	Natural logarithm of ECP value
Eosinophil.Abs.Value[2]	Numeric	Absolute eosinophil count (1/nL)
Eosinophil.Perc.Value[2]	Numeric	Eosinophils as percentage of leukocytes
pID	Integer	Irreversible hashed patient identifier (one-way hash; non-retraceable)
daytime.dec	Numeric	Sampling time (clock time, 24 h format rounded to one decimal place)
multiple columns with 3- or 4-letter ICD-10 codes	Logical	Diagnosis indicator (TRUE/FALSE), lifetime occurrence
multiple columns with 2-letter ICD-10 codes	Logical	Diagnosis indicator (TRUE/FALSE), lifetime occurrence
HAE.C1INH	Logical	HAE type 1/2 indicator (TRUE/FALSE), lifetime occurrence
HAE.nC1INH	Logical	HAE with normal C1-INH (type 3), lifetime occurrence
AAE.URT	Logical	Mast-cell-mediated angioedema (urticaria-associated), lifetime occurrence

[1] only available in Raw_ECP_database.csv

[2] only available in Raw_Eos_database.csv

Supplementary Figure legends

Supplementary Figure 1 – Power and Bayesian assurance calculation

The figure summarizes the statistical power and Bayesian assurance for detecting differences in log-transformed eosinophilic cationic protein (ECP) levels between patients with hereditary angioedema (HAE; $N = 48$) and non-HAE controls. Because classical power and assurance calculations cannot reflect the complexity of the full weighted model, we used pragmatic planning assumptions: a commonly used 1:3 case-control ratio was chosen, as increasing the number of controls beyond this point yields only minimal gains in power. The only data-derived quantity included in the calculations was the empirical standard deviation of log-ECP values. **Left panel:** Bayesian assurance across effect sizes (fold changes on the log-ECP scale) and prior widths of the HAE effect size (τ_{design}). **Right panel:** Frequentist power curves for a two-sample Student's t-test across plausible effect sizes (*Cohen's d*). These analyses provide a

simplified classical and Bayesian perspective on the expected sensitivity to detect clinically relevant elevations in ECP levels.

Supplementary Figure 2 – Eosinophilic cationic protein levels are increased in hereditary angioedema types 1 and 2

This figure is constructed in the same way as Figure 3 in the main manuscript but restricted to patients with HAE types 1 and 2. Control weights were recalculated for this subgroup to maintain covariate balance consistent with the main analysis. The increase in ECP levels observed in the overall HAE cohort is reproduced within this subgroup.

Supplementary Figure 3 – Eosinophilic cationic protein levels are increased in hereditary angioedema type 3

This figure is constructed in the same way as Figure 3 in the main manuscript but restricted to patients with HAE type 3. Control weights were recalculated for this subgroup to maintain covariate balance consistent with the main analysis. The pattern of increased ECP levels is again reproduced and appears even more pronounced than in HAE types 1 and 2.

Supplementary Figure 4 – Eosinophilic cationic protein levels are only minimally increased in mast-cell-mediated angioedema

This figure is constructed in the same way as Figure 3 in the main manuscript but restricted to patients with mast-cell-mediated acquired angioedema (AAE-URT). Control weights were recalculated for this subgroup to maintain covariate balance. ECP levels are significantly higher compared with counterfactual patients without angioedema, but the effect is much weaker than in HAE (fold change $\approx$ 1.15).

Supplementary Figure 5 – Visualization of raw ECP data

Combined beeswarm and weighted boxplots of raw log-transformed eosinophilic cationic protein (ECP) measurements for (**A**) HAE types 1 and 2 vs. controls, (**B**) HAE type 3 vs. controls, and (**C**) mast-cell-mediated acquired angioedema (AAE-URT). The ATT weights used in the subgroup models of **Supplementary Figures 2–4** are visualized through point opacity, following the conventions of **Figure 2** in the main text. For each group, the number of measurements, the sum of ATT weights, the effective sample size (ESS), and the weighted median with weighted 0.025 and 0.975 quantiles are shown. All boxplots incorporate ATT weighting to ensure consistency with the causal modelling framework.

Supplementary Figure 6 – Eosinophilic cationic protein levels are increased in females with hereditary angioedema

This figure is constructed in the same way as Figure 3 in the main manuscript but restricted to female patients and controls. Control weights were recalculated for this subgroup to maintain

covariate balance consistent with the main analysis. The increase in ECP levels observed in the overall HAE cohort is reproduced within this subgroup.

Supplementary Figure 7 – Eosinophilic cationic protein levels are not significantly increased in males with hereditary angioedema

This figure is constructed in the same way as Figure 3 in the main manuscript but restricted to male patients and controls. Control weights were recalculated for this subgroup to maintain covariate balance consistent with the main analysis. Point estimates are directionally consistent with the overall HAE cohort, but confidence intervals overlap the null, indicating no statistically significant increase.

Supplementary Figure 8 – Sex stratified visualization of raw ECP and eosinophil counts data

Combined beeswarm and weighted boxplots of raw log-transformed $[log(x)]$ eosinophilic cationic protein (ECP) measurements in (**A**) female and (**B**) male HAE patients vs. controls, and absolute eosinophil counts in (**C**) female and (**D**) male HAE patients vs. controls, transformed using the inverse hyperbolic sine $[asinh(x\ /\ c)]$. The ATT weights used in the subgroup models of **Supplementary Figures 6-7** are visualized through point opacity, following the conventions of **Figure 2** in the main text. For each group, the number of measurements, the sum of ATT weights, the effective sample size (ESS), and the weighted median with weighted 0.025 and 0.975 quantiles are shown. All boxplots incorporate ATT weighting to ensure consistency with the causal modelling framework.

Supplementary Figure 9 – Eosinophilic cationic protein levels are increased in females with hereditary angioedema type 1/2

This figure is constructed in the same way as Figure 3 in the main manuscript but restricted to female HAE type 1/2 patients and controls. Control weights were recalculated for this subgroup to maintain covariate balance consistent with the main analysis. The increase in ECP levels observed in the overall HAE cohort is reproduced within this subgroup.

Supplementary Figure 10 – Eosinophilic cationic protein levels are not significantly increased in males with hereditary angioedema type 1/2

This figure is constructed in the same way as Figure 3 in the main manuscript but restricted to male HAE type 1/2 patients and controls. Control weights were recalculated for this subgroup to maintain covariate balance consistent with the main analysis. Point estimates are directionally consistent with the overall HAE cohort, but confidence intervals overlap the null, indicating no statistically significant increase.

Supplementary Figure 11 – Sex-stratified visualization of raw ECP and eosinophil counts data in hereditary angioedema type 1/2 and controls

Combined beeswarm and weighted boxplots of raw log-transformed [$log(x)$] eosinophilic cationic protein (ECP) measurements in (**A**) female and (**B**) male HAE type 1/2 patients vs. controls, and absolute eosinophil counts in (**C**) female and (**D**) male HAE type 1/2 patients vs. controls, transformed using the inverse hyperbolic sine [$asinh(x\ /\ c)$]. The ATT weights used in the subgroup models of **Supplementary Figures 6-7** are visualized through point opacity, following the conventions of **Figure 2** in the main text. For each group, the number of measurements, the sum of ATT weights, the effective sample size (ESS), and the weighted median with weighted 0.025 and 0.975 quantiles are shown. All boxplots incorporate ATT weighting to ensure consistency with the causal modelling framework.

Supplementary Figure 12 – Absolute eosinophil counts in hereditary angioedema types 1 and 2

This figure mirrors the structure of **Supplementary Figure 2** but displays absolute eosinophil counts instead of ECP levels. The analysis is restricted to patients with HAE types 1 and 2, with control weights recalculated for this subgroup. The pattern observed in the overall cohort persists: HAE types 1 and 2 do not affect absolute eosinophil counts in peripheral blood.

Supplementary Figure 13 – Absolute eosinophil counts in hereditary angioedema type 3

Analogous to **Supplementary Figure 3**, this figure displays absolute eosinophil counts for patients with HAE type 3. Control weights were recalculated for this subgroup. As demonstrated for the total HAE cohort and the HAE type 1 and 2 sub-cohort, absolute eosinophil counts are not affected by HAE type 3.

Supplementary Figure 14 – Absolute eosinophil counts in mast-cell-mediated angioedema

This figure corresponds to the structure of Supplementary Figure 4, now showing absolute eosinophil counts in patients with mast-cell-mediated acquired angioedema (AAE-URT). Like HAE and HAE sub-cohorts, mast-cell-mediated acquired angioedema does not affect eosinophil numbers.

Supplementary Figure 15 – Raw data visualization for absolute eosinophil counts

Combined beeswarm and weighted boxplots of raw absolute eosinophil counts for (A) HAE types 1 and 2 vs. controls, (B) HAE type 3 vs. controls, and (C) mast-cell-mediated angioedema (AAE-URT). Scale transformation, ATT weights, point opacities, and the computation of weighted medians, 0.025–0.975 quantiles, and effective sample sizes follow the same conventions as in **Figure 2** and **Supplementary Figure 5**.

Supplementary Figure 16 – Absolute eosinophil counts are unchanged in females with hereditary angioedema

This figure is constructed in the same way as Figure 4 in the main manuscript but restricted to female patients and controls. Control weights were recalculated for this subgroup to maintain covariate balance consistent with the main analysis. The absence of a measurable effect of HAE on absolute eosinophil counts is reproduced within this subgroup.

Supplementary Figure 17 – Absolute eosinophil counts are unchanged in males with hereditary angioedema

This figure is constructed in the same way as Figure 4 in the main manuscript but restricted to male patients and controls. Control weights were recalculated for this subgroup to maintain covariate balance consistent with the main analysis. The absence of a measurable effect of HAE on absolute eosinophil counts is reproduced within this subgroup.

Supplementary Figure 18 – Absolute eosinophil counts are unchanged in females with hereditary angioedema type 1/2

This figure is constructed in the same way as Figure 4 in the main manuscript but restricted to female HAE type 1/2 patients and controls. Control weights were recalculated for this subgroup to maintain covariate balance consistent with the main analysis. The absence of a measurable effect of HAE on absolute eosinophil counts is reproduced within this subgroup.

Supplementary Figure 19 – Absolute eosinophil counts are unchanged in males with hereditary angioedema type 1/2

This figure is constructed in the same way as Figure 4 in the main manuscript but restricted to male HAE type 1/2 patients and controls. Control weights were recalculated for this subgroup to maintain covariate balance consistent with the main analysis. The absence of a measurable effect of HAE on absolute eosinophil counts is reproduced within this subgroup.
