# Supplementary material for: Eosinophilic inflammation in hereditary angioedema: a single-center real-world retrospective chart review study
Source: Front Immunol. 2026 Feb 17;17:1754405. doi: 10.3389/fimmu.2026.1754405 (PMC12953394; doi:10.3389/fimmu.2026.1754405)
Supplement: Supplementary file 3 [file DataSheet3.zip › Supplementary Figure 1 - PowerAssuranceSupplement.pdf]

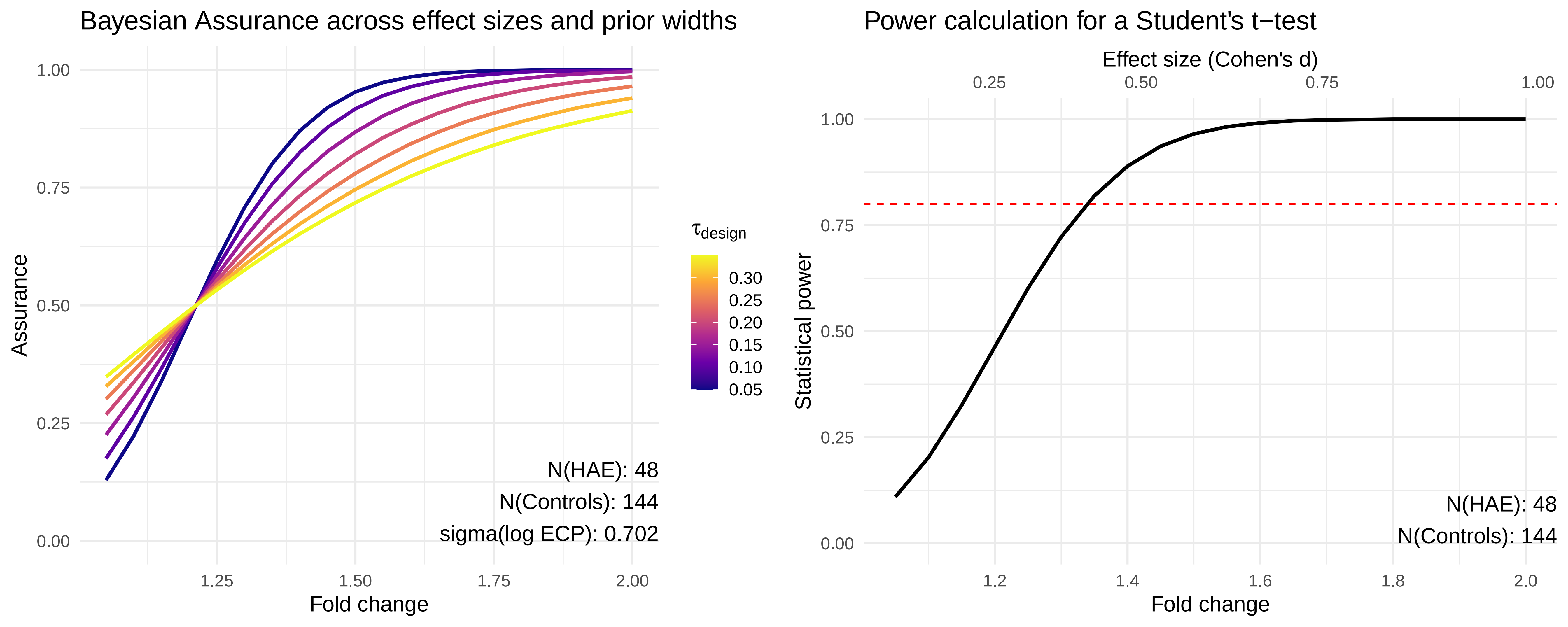
Bayesian Assurance across effect sizes and prior widths
Assurance
Fold change
1.00
0.75
0.50
0.25
0.00
1.25
1.50
1.75
2.00
τ_{design}
0.30
0.25
0.20
0.15
0.10
0.05
N(HAE): 48
N(Controls): 144
sigma(log ECP): 0.702
Power calculation for a Student's t−test
Effect size (Cohen's d)
0.25
0.50
0.75
1.00
Statistical power
Fold change
1.2
1.4
1.6
1.8
2.0
N(HAE): 48
N(Controls): 144
