# Supplementary figures and images for: Eosinophilic inflammation in hereditary angioedema: a single-center real-world retrospective chart review study
Source: Front Immunol. 2026 Feb 17;17:1754405. doi: 10.3389/fimmu.2026.1754405 (PMC12953394; doi:10.3389/fimmu.2026.1754405)

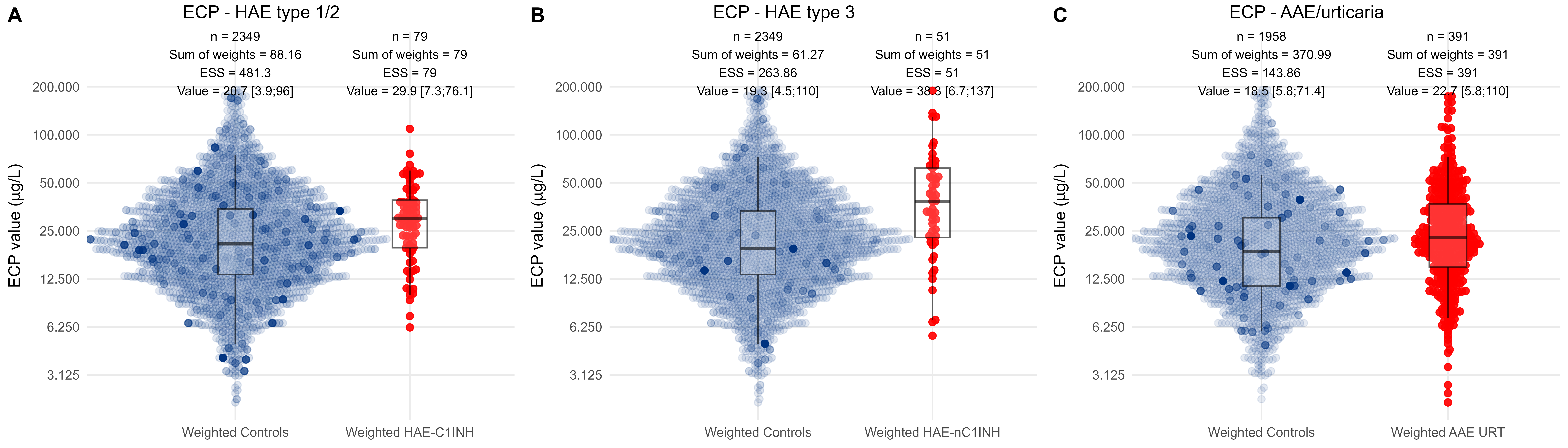

Supplement: Supplementary file 3 [file DataSheet3.zip › supplementary-figure-5---raw_supplement_ecp_qc_20260112T222639.tiff]

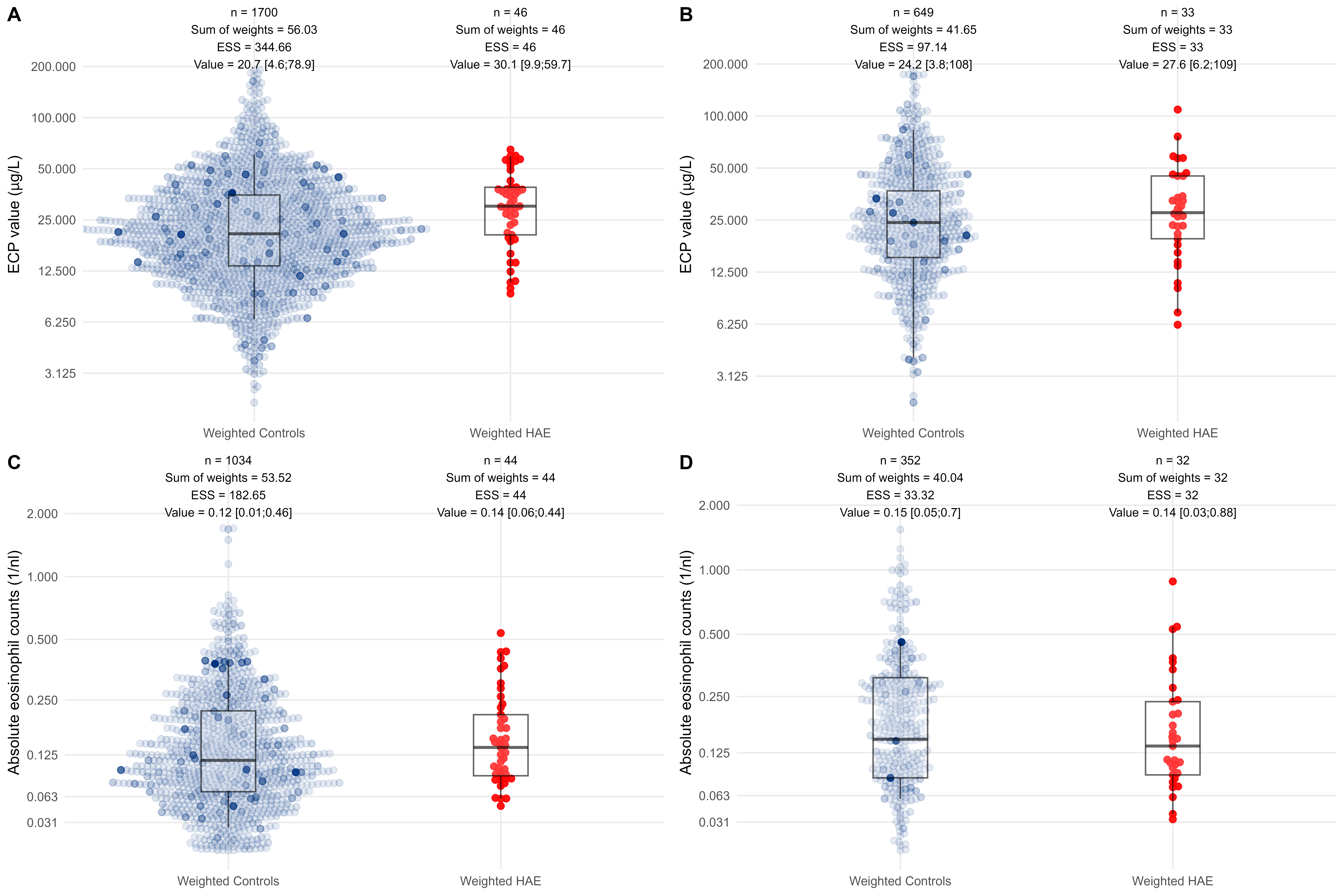

Supplement: Supplementary file 4 [file DataSheet4.zip › supplementary-figure-11---male_female_hae12_raw_plot_qc_20260112T222636.tiff]

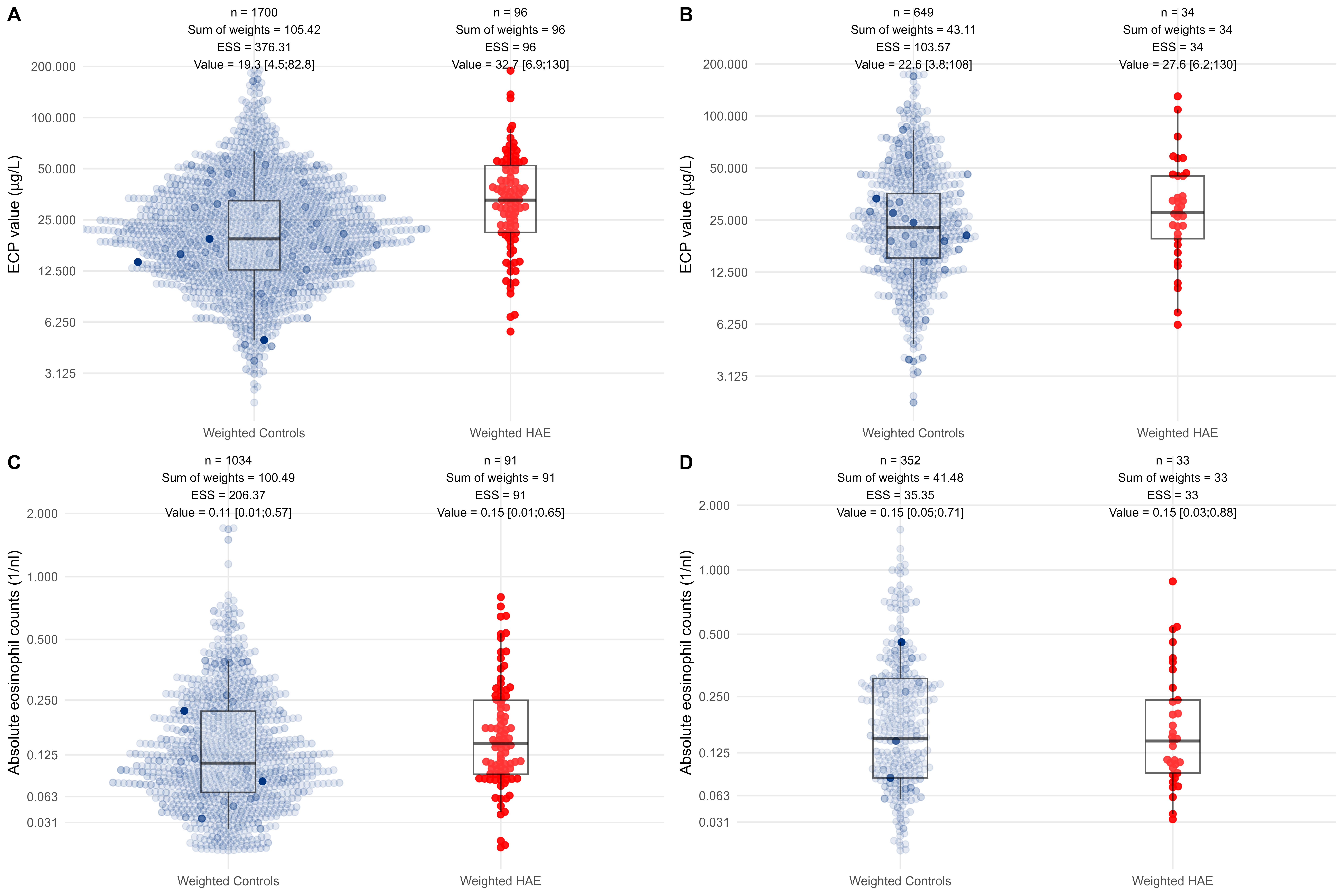

Supplement: Supplementary file 4 [file DataSheet4.zip › supplementary-figure-8---male_female_raw_plot_qc_20260112T222607.tiff]

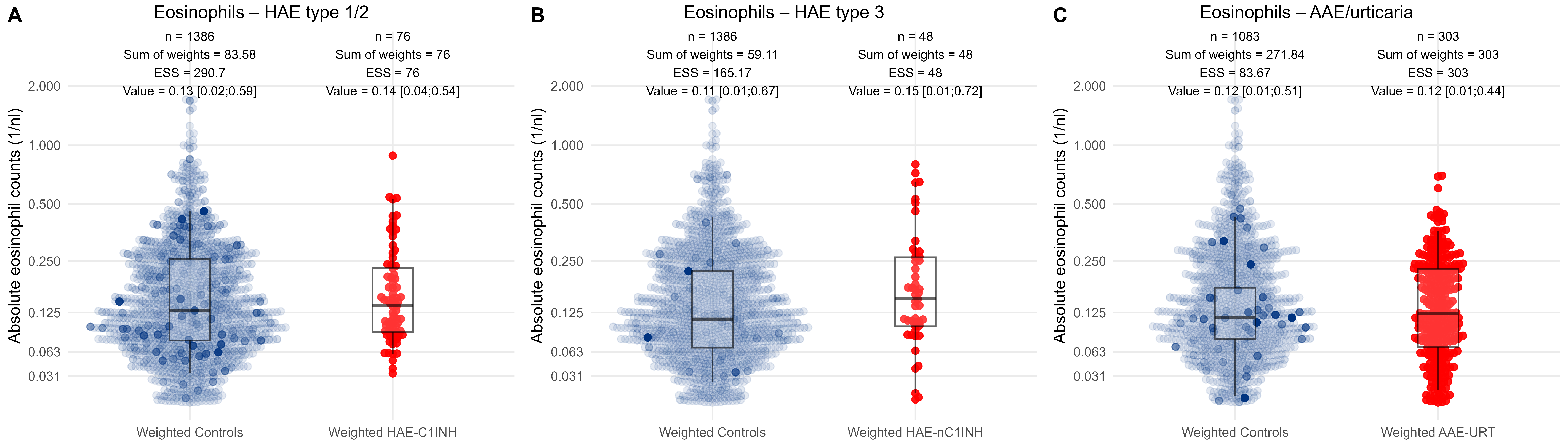

Supplement: Supplementary file 5 [file DataSheet5.zip › supplementary-figure-15--raw_supplement_eosinophils_qc_20260112T222641.tiff]
