# Supplementary material for: Eosinophilic inflammation in hereditary angioedema: a single-center real-world retrospective chart review study
Source: Front Immunol. 2026 Feb 17;17:1754405. doi: 10.3389/fimmu.2026.1754405 (PMC12953394; doi:10.3389/fimmu.2026.1754405)
Supplement: Supplementary file 6 [file Table1.pdf]

Supplementary Table 1. Demographic and clinical characteristics of patient cohorts (stratified by sex)

Characteristic		Males				Females			
		ECP measurement		Eosinophil measurement		ECP measurement		Eosinophil measurement	
		HAE[1]	Non-HAE[1]	HAE[1]	Non-HAE[1]	HAE[1]	Non-HAE[1]	HAE[1]	Non-HAE[1]
Demo-graphics	Patients (N)	17	528	16	268	31	1352	31	804
	Age (years, mean ± SD)	44.5 ± 20.9	45.6 ± 18.0	45.6 ± 21.0	46.5 ± 17.8	40.8 ± 15.7	45.8 ± 16.1	40.8 ± 15.7	46.1 ± 15.7
	Age (years, median [IQR])	47.0 [32.0; 55.0]	46.0 [31.0; 60.0]	49.5 [33.5; 55.8]	48.0 [31.0; 61.0]	46.0 [25.0; 51.5]	45.0 [34.0; 58.0]	46.0 [25.0; 51.5]	46.0 [34.0; 58.0]
AAE/HAE type	HAE-C1INH (HAE1/2)	16	0	15	0	16	0	16	0
	HAE-nC1INH (HAE3)	1	0	1	0	15	0	15	0
	Mast-cell mediated angioedema (AAE-URT)	1	74	1	57	5	212	5	169
Comorbidities (ICD10 3 letter codes)	C96 – Other and unspecified malignant neoplasms of lymphoid, hematopoietic and related tissue	0 (0.0%)	1 (0.2%)	0 (0.0%)	0 (0.0%)	0 (0.0%)	12 (0.9%)	0 (0.0%)	10 (1.2%)
	D47 — Other neoplasms of uncertain or unknown behaviour of lymphoid, haematopoietic and related tissue	0 (0.0%)	24 (4.5%)	0 (0.0%)	20 (7.5%)	2 (6.5%)	94 (7.0%)	2 (6.5%)	78 (9.7%)
	H10 — Conjunctivitis	0 (0.0%)	9 (1.7%)	0 (0.0%)	2 (0.7%)	0 (0.0%)	40 (3.0%)	0 (0.0%)	12 (1.5%)
	J30 — Vasomotor and allergic rhinitis	1 (5.9%)	171 (32.4%)	0 (0.0%)	65 (24.3%)	5 (16.1%)	399 (29.5%)	5 (16.1%)	215 (26.7%)
	J32 — Chronic sinusitis	0 (0.0%)	13 (2.5%)	0 (0.0%)	13 (4.9%)	0 (0.0%)	19 (1.4%)	0 (0.0%)	18 (2.2%)
	J33 — Nasal polyp	0 (0.0%)	6 (1.1%)	0 (0.0%)	4 (1.5%)	0 (0.0%)	10 (0.7%)	0 (0.0%)	10 (1.2%)
	J45 — Asthma	0 (0.0%)	22 (4.2%)	0 (0.0%)	11 (4.1%)	0 (0.0%)	68 (5.0%)	0 (0.0%)	39 (4.9%)
	L20 — Atopic dermatitis	0 (0.0%)	30 (5.7%)	0 (0.0%)	13 (4.9%)	1 (3.2%)	103 (7.6%)	1 (3.2%)	55 (6.8%)
	L23 — Allergic contact dermatitis	0 (0.0%)	23 (4.4%)	0 (0.0%)	9 (3.4%)	0 (0.0%)	71 (5.3%)	0 (0.0%)	32 (4.0%)
	L27 — Dermatitis due to substances taken internally	0 (0.0%)	4 (0.8%)	0 (0.0%)	1 (0.4%)	0 (0.0%)	8 (0.6%)	0 (0.0%)	3 (0.4%)
	L29 — Pruritus	0 (0.0%)	5 (0.9%)	0 (0.0%)	5 (1.9%)	0 (0.0%)	13 (1.0%)	0 (0.0%)	9 (1.1%)
	L30 — Other dermatitis	0 (0.0%)	23 (4.4%)	0 (0.0%)	11 (4.1%)	2 (6.5%)	17 (1.3%)	2 (6.5%)	12 (1.5%)
	L50 — Urticaria	0 (0.0%)	123 (23.3%)	0 (0.0%)	97 (36.2%)	6 (19.4%)	366 (27.1%)	6 (19.4%)	293 (36.4%)
	T63 — Toxic effect of contact with venomous animals	0 (0.0%)	39 (7.4%)	0 (0.0%)	8 (3.0%)	0 (0.0%)	50 (3.7%)	0 (0.0%)	22 (2.7%)
	T78 — Adverse effects, not elsewhere classified	1 (5.9%)	198 (37.5%)	1 (6.2%)	109 (40.7%)	5 (16.1%)	552 (40.8%)	5 (16.1%)	359 (44.7%)
	Z88 — Personal history of allergy to drugs, medicaments and biological substances	0 (0.0%)	52 (9.8%)	0 (0.0%)	21 (7.8%)	5 (16.1%)	206 (15.2%)	5 (16.1%)	101 (12.6%)
Lab. values	ECP measurements (N)	34	649	33	352	96	1700	91	1034
	Avg. measurements per patient	2.00	1.23	2.06	1.31	3.10	1.26	2.94	1.29
	ECP (median [IQR][min;max])	28.4 [19.8; 45.0] [6.2; 130]	24.5 [15.1; 42.2] [2.2; 183]	27.6 [19.5; 45.0] [6.2; 130]	26.0 [15.7; 47.3] [2.2; 183]	32.9 [21.2; 52.4] [5.5; 189]	21.1 [13.1; 33.9] [2.1; 191]	32.6 [21.2; 48.4] [5.5; 189]	21.1 [12.9; 33.7] [2.1; 191]
	Eosinophils absolute measurements (N)			33	352			91	1034
	Eosinophils absolute (median [IQR] [min;max])			0.14 [0.09; 0.24] [0.03; 0.88]	0.19 [0.10; 0.30] [0; 1.54]			0.14 [0.09; 0.23] [0;0.79]	0.11 [0.06; 0.21] [0; 1.70]
	Eosinophils percent measurements (N)			33	352			91	1034
	Eosinophils percent (median [IQR] [min;max])			2.10 [1.40; 3.90] [0.6; 10.6]	2.80 [1.58; 4.70] [0; 20.9]			1.80 [1.30; 2.95] [0; 9.8]	1.70 [1.00; 3.00] [0; 17.0]

1 If not indicated otherwise: Number (N) of patients with the respective characteristic, and percentage of total (%).

2 Reference ranges were 0 - 13.3 µg/L for serum ECP, 0.02 - 0.75 /nL for absolute eosinophil counts, and 0.5 -5.5 % for relative eosinophil counts.
